# Modeling the Pro-inflammatory Tumor Microenvironment in Acute Lymphoblastic Leukemia Predicts a Breakdown of Hematopoietic-Mesenchymal Communication Networks

**DOI:** 10.3389/fphys.2016.00349

**Published:** 2016-08-19

**Authors:** Jennifer Enciso, Hector Mayani, Luis Mendoza, Rosana Pelayo

**Affiliations:** ^1^Oncology Research Unit, Mexican Institute for Social SecurityMexico City, Mexico; ^2^Biochemistry Sciences Program, Universidad Nacional Autónoma de MexicoMexico City, Mexico; ^3^Departamento de Biología Molecular y Biotecnología, Instituto de Investigaciones Biomédicas, Universidad Nacional Autónoma de MexicoMexico City, Mexico

**Keywords:** cancer systems biology, acute lymphoblastic leukemia, tumor microenvironment, CXCL12, pro-inflammatory bone marrow, early hematopoiesis, network modeling, dynamical systems

## Abstract

Lineage fate decisions of hematopoietic cells depend on intrinsic factors and extrinsic signals provided by the bone marrow microenvironment, where they reside. Abnormalities in composition and function of hematopoietic niches have been proposed as key contributors of acute lymphoblastic leukemia (ALL) progression. Our previous experimental findings strongly suggest that pro-inflammatory cues contribute to mesenchymal niche abnormalities that result in maintenance of ALL precursor cells at the expense of normal hematopoiesis. Here, we propose a molecular regulatory network interconnecting the major communication pathways between hematopoietic stem and progenitor cells (HSPCs) and mesenchymal stromal cells (MSCs) within the BM. Dynamical analysis of the network as a Boolean model reveals two stationary states that can be interpreted as the intercellular contact status. Furthermore, simulations describe the molecular patterns observed during experimental proliferation and activation. Importantly, our model predicts instability in the CXCR4/CXCL12 and VLA4/VCAM1 interactions following microenvironmental perturbation due by temporal signaling from Toll like receptors (TLRs) ligation. Therefore, aberrant expression of NF-κB induced by intrinsic or extrinsic factors may contribute to create a tumor microenvironment where a negative feedback loop inhibiting CXCR4/CXCL12 and VLA4/VCAM1 cellular communication axes allows for the maintenance of malignant cells.

## Introduction

Cancer is currently considered as a global child health priority (Gupta et al., 2014). The application of effective treatments to decrease overall childhood cancer mortality requires a comprehensive understanding of its origins and pathobiology, along with accurate diagnosis and early identification of high-risk groups (reviewed in Vilchis-Ordoñez et al., 2016). Strikingly, the clinical, molecular and biological heterogeneity of malignant diseases indicating an unsuspected multiclonal diversity has highlighted their complexity and the uncertainty of their cell population dynamics. Novel theoretical and experimental integrative strategies have changed our perspective of cancer, from a hierarchical, deterministic and unidirectional process to a multi-factorial network where genetics interacts with micro and macro environmental cues that contribute to the etiology and maintenance of tumor cells (Notta et al., 2011; Davila-Velderrain et al., 2015; Tomasetti and Vogelstein, 2015). Furthermore, stochastic effects associated with the number of stem cell divisions have been proposed as major contributors, often even more significant than hereditary or external factors (Tomasetti and Vogelstein, 2015).

B-cell acute lymphoblastic leukemia (B-ALL) is largely the result of a growing number of cooperating genetic and epigenetic aberrations that corrupt hematopoietic developmental pathways and ultimate lead to uncontrolled production of malignant B lymphoid precursor cells within the bone marrow (BM) (Pelayo et al., 2012; Purizaca et al., 2012). Leukemic cell infiltration and treatment failure worsen the outcome of the disease and remain the foremost cause of relapse. Recent advances suggest the ability of leukemia initiating cells to create abnormal BM microenvironments, promoting high proliferation and early differentiation arrest at the expense of normal cell fate decisions (Colmone et al., 2008; Raaijmakers, 2011; Vilchis-Ordoñez et al., 2015). Intrinsic damage and/or remodeling of cell compartments that shape the distinct BM niches may account to microenvironmental regulation of quiescence, proliferation, differentiation and blastic cell migration. Leukemic cells compete for niche resources with their normal hematopoietic counterparts (Wu et al., 2009), culminating in the displacement of the latter, as observed in xenotransplantation mice models (Colmone et al., 2008). Moreover, the marrow microenvironment provides leukemic precursors with dynamic interactions and regulatory signals that are essential for their maintenance, proliferation and survival. Although, the underlying molecular mechanisms are poorly defined, these niches protect tumor cells from chemotherapy-induced apoptosis, showing a new perspective on the evolution of chemoresistance (Ayala et al., 2009: Shain et al., 2015; Tabe and Konopleva, 2015), and emphasizing the need for new models that theoretically or experimentally replicate the interplay between tumor and stromal cells under normal and pathological settings.

As suggested by our previous findings, ALL lymphoid precursors have the ability of responding to pathogen- or damage- associated molecular patterns via Toll-like receptor signaling by secreting soluble factors and altering their differentiation potentials (Dorantes-Acosta et al., 2013). The resulting pro-inflammatory microenvironment may expose them to prolonged proliferation, contributing tumor maintenance in a self-sustaining way while prompting the NF-κB-associated proliferation of normal progenitor cells (Vilchis-Ordoñez et al., 2015, 2016). Some hematopoietic growth factors and pro-inflammatory cytokines, including granulocyte-colony stimulating factor (G-CSF), IFNα, IL-1α, IL-1β, IL-7, and TNFα were highly produced by ALL cells from a conspicuous group of patients co-expressing myeloid markers (Vilchis-Ordoñez et al., 2015). Of note, mesenchymal stromal cells (MSCs) from ALL BM have shown atypical production of pro-inflammatory factors whereas disruption of the major cell communication pathway is apparent by detriment of CXCL12 expression and biological function (Geay et al., 2005; Colmone et al., 2008; van den Berk et al., 2014).

Considering that the CXCL12/CXCR4 axis constitutes the most critical component of the perivascular and reticular BM niches supporting the hematopoietic stem and progenitor cells (HSPCs) differentiation and maintenance within the BM, as well as the early steps of B cell development (Ma et al., 1998; Tokoyoda et al., 2004; Sugiyama et al., 2006; Greenbaum et al., 2013), an obstruction of the HSPC-MSC interaction may have substantial implications in the overall stability of these processes. Whether the inflammation-derived signals provide a mechanism for leukemic cells to survive, to induce changes in lineage cell fate decisions, or to prompt niche remodeling in leukemia settings, are currently topical questions.

Mathematical model strategies have become powerful approaches to complex biological systems and may contribute to unravel the hematopoietic-microenvironment interplay that facilitates tumor cells prevalence (Altrock et al., 2015; Enciso et al., 2015). Through continuous dynamic modeling with differential equations we have learned seminal aspects of multi-compartment and multi-clonal behavior of leukemic cell populations (Stiehl and Marciniak-Czochra, 2012; Enciso et al., 2015), leading to novel proposals on disease development driven by unbalanced competition between normal and pre-leukemic cells (Swaminathan et al., 2015). Both stochastic and deterministic models have been useful to simulate cell fate decisions and predict clonal evolution (reviewed in Enciso et al., 2015). Certainly, incorporating tumor microenvironment in cancer modeling is expected to change our vision of biochemical interactions in niche remodeling-dependent hematopoietic growth, as recently demonstrated for myeloma disease (Coelho et al., 2016).

By developing and simulating a dynamic Boolean system, we now investigate the biological consequences of microenvironmental perturbation due by temporal TLR signaling on crucial communication networks between stem/progenitor cells (HSPCs) and MSCs in ALL. We propose that NF-κB dependent tumor-associated inflammation co-participate in malignant progression concomitant to normal hematopoietic failure through disruption of CXCL12/CXCR4 and VLA4/VCAM-1 communication axes.

## Materials and methods

### Manual curation strategy

Based on the crucial and unique role of the CXCL12/CXCR4 axis in the regulation of maintenance, biological activity, and niche communication-derived cell fate decisions of seminal cells, including pluripotent embryonic stem cells and multipotent hematopoietic stem cells, construction and updating of molecular interactions of relevance involved careful manual curation of primary hematopoietic cell research. Moreover, of special interest was the attention to the hematopoietic malignancies, which in contrast to solid tumors, display a distinct CXCL12-mediated microenvironmental behavior. Thus, although the modeled signaling pathways could be considered generic to all tissues, the organ, stage of cell differentiation and surrounding microenvironment may influence the net result of interactions. Taking into account this considerations, most published work that has been used for the reconstruction of our proposed model, include data from molecular interactions in HSPCs. Some of the interactions have been reported in a number of different tissues and predicted to be conserved in the hematopoietic system. Finally, as there is not enough data to model hematopoietic-microenvironment restricted to *Homo sapiens* and some interactions might be crucial for the molecular connectivity of the model, we have used information from different species when needed. A detailed referencing of all reports used for the model reconstruction is provided as Supplemental Material (Tables S1, S2, and reference list).

### Molecular basis for the network reconstruction

The connectivity among key molecules involved in the communication between HSPCs and MSCs within the BM was inferred through the curated experimental literature. Specifically, we were interested in recovering the network components, their interactions, and the nature of the interactions (activation/positive or inactivation/negative). The resulting general network incorporates transcriptional factors, kinases, membrane receptors, interleukines, integrins, growth factors, and chemokines from Homo sapiens and Mus musculus species. Importantly, to simplify the modeling process, some groups of molecules were considered as single functional modules, thus encompassing a series of sequential steps that lead to the activation or inactivation of a certain node (e.g., PI3K/Akt). The following paragraphs summarize the principle evidence used to reconstruct the HSPC-MSC network and infer the logical rules for computational simulation of the system as a discrete dynamical model. A detailed referencing is provided as Supplemental Material (Tables S1, S2, and reference list).

The CXCR4/CXCL12 chemokine pathway was considered as the central axis for the network construction considering its essential role in homeostasis maintenance (Sugiyama et al., 2006; Tzeng et al., 2011) and B lineage support (Ma et al., 1998; Tokoyoda et al., 2004). Furthermore, recent observations suggest that this axis is disrupted by up-stream molecular deregulations both in MSC and leukemic blasts harvested from ALL patients, affecting the maintenance of hematopoietic cells within their regulatory niches (Geay et al., 2005; Colmone et al., 2008; van den Berk et al., 2014). Besides the well-studied CXCR4/CXCL12 chemotactic interaction, CXCR4 activation increases the affinity between vascular cellular adhesion molecule-1 (VCAM-1) expressed on the surface of MSC and its receptor VLA-4 on HSPC. Both pathways, CXCR4/CXCL12 and VLA-4/VCAM-1, are known to play coordinately a central role in HSPC migration, engraftment and retention within the BM (Peled et al., 2000; Ramirez et al., 2009), converge in triggering the PI3K/Akt and ERK signals, and share common up-stream regulators involving molecular factors guiding inflammatory responses.

As mentioned in the Introduction, recent evidence indicates the secretion of high levels of pro-inflammatory cytokines by a conspicuous group of ALL patients (Vilchis-Ordoñez et al., 2015), thereby presumably contributing to remodeling of the normal hematopoietic microenvironment (Colmone et al., 2008). Of note, interleukin-1α (IL-1α) and IL-1β, which were substantially elevated, play an amplification role on inflammation increasing the expression of other cytokines, like G-CSF (Majumdar et al., 2000; Allakhverdi et al., 2013), and setting a positive feedback loop with the PI3K co-activation of NF-κB (Reddy et al., 1997; Sizemore et al., 1999; Carrero et al., 2012; Bektas et al., 2014). IL-1 and G-CSF, inhibit directly and indirectly the CXCR4/CXCL12 axis. G-CSF negatively regulates CXCL12 transcription and increases the secretion of matrix metalloproteinase-9, showing the ability to degrade both CXCL12 (Lévesque et al., 2003; Semerad et al., 2005; Christopher et al., 2009; Day et al., 2015) and CXCR4 (Lévesque et al., 2003). Moreover, G-CSF promotes up-regulation of Gfi1 that at the time inhibits the transcription of CXCR4 (Zhuang et al., 2006; De La Luz Sierra et al., 2007; de la Luz Sierra et al., 2010). Thus, by considering this information from experimental data, we have included IL-1 and G-CSF as key elements of the BM microenvironment in the HSPC-MSC communication network.

In concordance, we incorporated as a “positive control condition” an input node representing the Toll-like receptor ligand (lTLR) lipopolysaccharide (LPS), that binds TLR4 and triggers the conventional and well-known NF-κB-dependent pro-inflammatory response, promoting, among other transcriptional targets, the transcription of pro-IL-1β (Jones et al., 2001; Tak and Firestein, 2001; Wang et al., 2002; Khandanpour et al., 2010; Higashikuni et al., 2013).

Downstream NF-κB, the expression of CXCR7 has been shown to be upregulated (Tarnowski et al., 2010), which in turn, down-regulates CXCR4 by heterodimerization, promoting its internalization and further degradation. In parallel, activated CXCR7 presents a higher affinity for CXCL12 and β-arrestin, reducing CXCR4 signaling in CXCR7 and CXCR4 expressing cells (Uto-Konomi et al., 2013; Coggins et al., 2014). However, CXCR7 is unable to couple with G-protein, transducing through recruitment of β-arrestin and leading to MAP kinases Akt and ERK activation (Tarnowski et al., 2010; Uto-Konomi et al., 2013; Torossian et al., 2014). As with CXCR4, CXCR7, and VLA-4 activation in HSPC, PI3K/Akt pathway is activated on HSPC and MSC, via G-CSF receptor signaling (Liu et al., 2007; Vagima et al., 2009; Ponte et al., 2012; Furmento et al., 2014), and after LPS stimulation (Guha and Mackman, 2002; Wang et al., 2009; McGuire et al., 2013). Apparently, PI3K/Akt acts at overlapping levels on the modulation of inflammation. On the one hand, it increases the production of IL-1 antagonist molecules (Williams et al., 2004; Molnarfi et al., 2005; Li and Smith, 2014) and inhibits secretion of mature IL-1β (Tapia-Abellán et al., 2014). On the other hand, it promotes nuclear translocation of the transcriptional factor Foxo3a (Brunet et al., 1999; Miyamoto et al., 2008; Park et al., 2008), down-regulating indirectly the transcription of antioxidant enzymes and enabling reactive oxygen species (ROS) accumulation, which in turn promotes maturation of pro-IL-1β and IL-1β secretion (Hsu and Wen, 2002; Yang et al., 2007; Gabelloni et al., 2013).

At the mesenchymal counterpart, in addition to a number of molecules participating in the MSC-subsystem sensitivity to microenvironmental cues, we incorporated an input node representing Gap-junction conformed by connexin-43 (Cx43) that mediates direct intercellular communication between mesenchymal cells. Strikingly, its integral activity as calcium channel conductor has been shown to be a potent positive regulator of CXCL12 transcription and secretion (Schajnovitz et al., 2011). Furthermore, Cx43 expression appears to be critically disregulated in the BM stromal cells from acute leukemia patients, suggesting an important role in the hypothetic disregulation of the hematopoietic-stromal intercellular communication (Liu et al., 2010; Zhang et al., 2012). The inclusion of GSK3β and β-catenin in both subsystems was relevant due to their roles as intermediates of signaling transduction and regulation of the main intracellular communication elements proposed for our network reconstruction. The model is available in XML format (GINML) on GINsim Model Repository page (http://ginsim.org/models_repository) (Chaouiya et al., 2012), under the title “HSPCs-MSCs. Communication pathways between Hematopoietic Stem Progenitor Cells (HSPCs) and MSCs.”

### Dynamical modeling of the HSPC-MSC network

For the computational modeling of the HSPC-MSC complex system, we followed the standard steps to convert it into a discrete dynamical system, as described by Albert and Wang (2009) and Assman and Albert (2009). The Boolean approach is useful when quantitative and detailed kinetic information is lacking. In such a case, each node of the network is represented as a binary element, allowed only to have an “active” (ON) or “inactive” (OFF) state, numerically represented by 1 and 0, respectively. The activation state of each node is dependent on the activation state of its regulators, as described by Boolean functions, also called logical rules. The classical Boolean operators employed in Boolean functions are AND (&), OR (|) and NOT (!). The AND operator is used to represent the requirement of the conjunction of two or more nodes participating in the regulation of a certain node (e.g., *VLA-4* = *CXCR4 & VCAM-1* representing that VLA-4 optimal activation requires its ligand VCAM-1 and the signaling due to CXCR4 activation). When there is more than one node able to regulate another, but only one of them is sufficient to exert the effect, the OR operator is applied (e.g., *PI3K/Akt* = *GCSF | ROS | TLR* representing that the activation of the G-CSF receptor, the increase of intracelular ROS concentration or the binding of a TLR ligand may activate PI3K/Akt signaling). Finally, the NOT operator represents repression of a node over another (e.g., *IL-1* = *(NF*-κ*B & ROS) & !PI3K/Akt* meaning that IL-1 requires the transcriptional activation of pro-IL-1 promoted by NF-κB and the post-transcriptional maturation mediated by ROS, but its signaling is inhibited by the presence of PI3K/Akt). Detailed compiling of reviewed references for the network reconstruction and the development of the logical rules can be found in Tables S1, S2.

Given that each node in the network has an activation state, then the general state of a network at a given time *t* can be represented by a vector of *n* elements, where *n* is the number of nodes in the network. For example, the vector (00000010000000000100001000), represents a network state where only the 7th, 18th, and 23rd elements are active. In our model, this particular state represents the pattern of activation where only GSK3B_H, GSK3B_M, and VCAM1_M are active. Now, since we are implementing a dynamical system, it is necessary to specify how the network may evolve from a time *t* to *t*+*1*.

There are two possible implementations to model the transition from one state of the network to another. On one side, the synchronous scheme update the activation state of all the nodes each time-step, assuming that all the biological processes involved in the model occur at similar time scales. And on the other side, asynchronous scheme update only one of the logical rules per time step, considering a more complex behavior of biological processes where molecular signaling is likely to change at different time points depending on the nature of the interaction (Albert and Wang, 2009). Either one or another update scheme, take an initial combination of the nodes (initial state) and update the logical rules successively through an established number of time steps or until an steady state or attractor is reached. Attractors may be of a single state (fixed point attractors) or a set of states (cyclic or complex attractors depending if they have one or more possible transition paths among their constituent states). The analysis of the nodes activation pattern in the attractors give the biological significance of the computational simulations of the models (Albert and Wang, 2009; Assman and Albert, 2009).

The dynamical behavior of the network was analyzed implementing the logical rules into BoolNet (R open-source package), and obtaining its attractors (stationary states) by applying asynchronous update strategies (Müssel et al., 2010). Under the asynchronous updating scheme, the simulation was performed using 50,000 random initial states, updating the network until either a fixed point attractor or a complex attractor was reached. Confidence of the model was tested through the simulation of all possible mutants (constitutive and null activation of every node) and the comparison of the resultant attractors with experimental reports about the biological effects *in vivo* or *in vitro* after the use of antagonists or the generation of knock-in and knock-out models.

### Dynamical multicellular approach

Assuming that every simulation beginning at a certain initial state of the network represents the dynamical profile of a single cell, Wu and collaborators proposed a “population-like” analysis for a discrete model (Wu et al., 2009). Similarly, we asynchronously ran the simulations of the network from 50,000 random initial states, and then updated for 2000 time-steps, followed by calculation of the average activation value from 50,000 simulations for each node in each time-step. Such data was plotted as multi-cellular average activation graphs. Furthermore, we evaluated the effect of a short (1 time-step) and a sustained (699 time-steps) temporary induction of lTLR in time-step 700 and 1400, and analyzed the dynamical effects in the wild type network and in some relevant mutant networks.

## Results

### Network reconstruction

**Figure 1:**
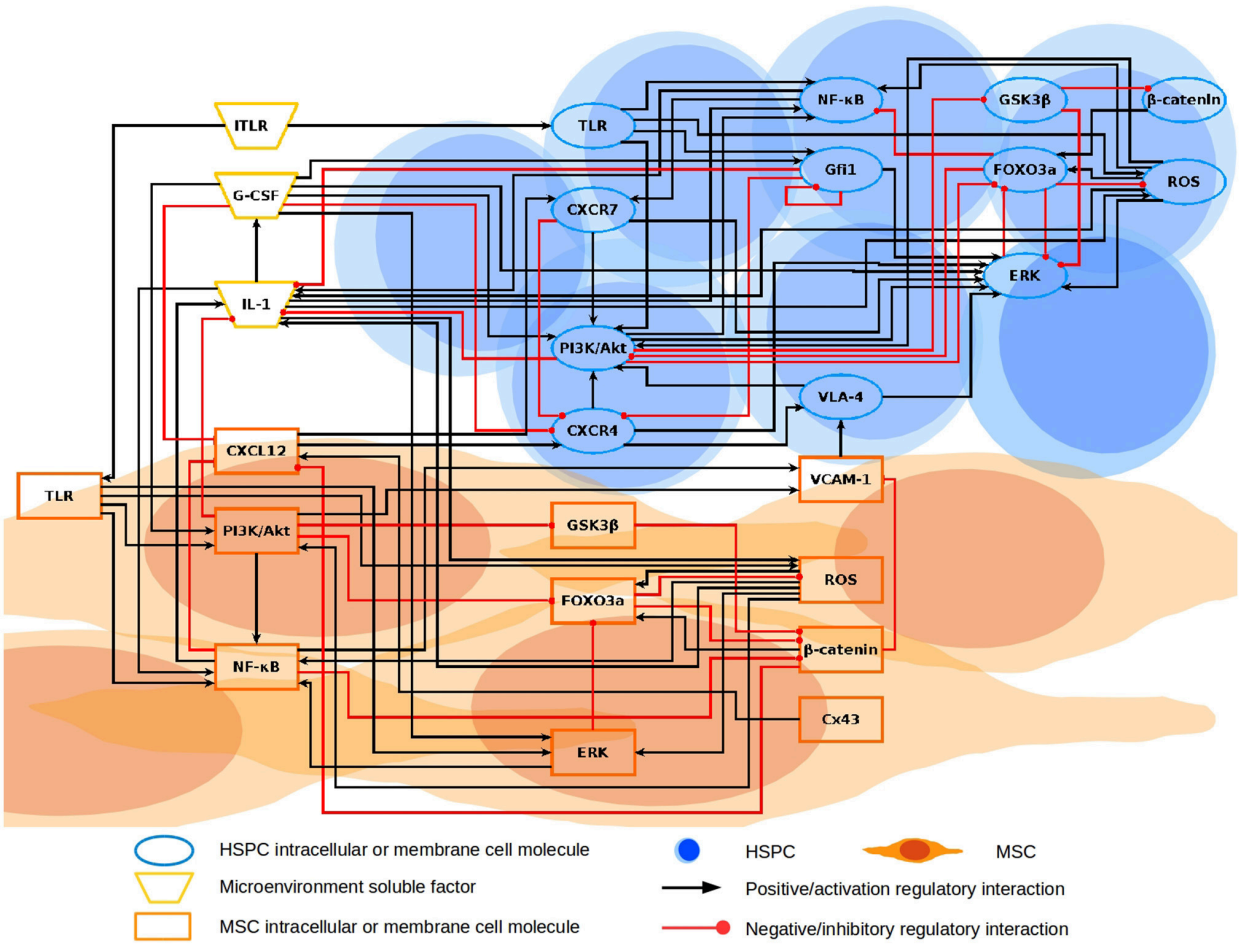
**Regulatory HSPC-MSC network**. The network is constituted by three compartments represented with different geometric shapes: HSPC, MSC, and microenvironmental soluble factors. HSPC and MSC have intracellular nodes regulating the response and expression of elements mediating the communication between them. CXCR4-CXCL12 and VLA-4/VCAM-1 axes are suggested to be the most crucial communicating elements. HSPC and MSC are both susceptible of TLR stimulation with lTLR input. HSPC, hematopoietic stem and progenitor cell; MSC, mesenchymal stromal cell.

The inferred HSPC-MSC network (Figure 1) constitutes the first attempt to model relevant interaction axes between undifferentiated hematopoietic cells and the BM microenvironment, that may approach us to a deeper understanding of the numerous molecular signals influencing the hematopoietic system regulation during normal and malignant processes. Our current ALL network has 26 nodes and 80 interactions. Among them, twelve nodes correspond to molecules that are expressed in HSPC and involved in intracellular signaling (PI3K/Akt, Gfi1, NF-κB, GSK3β, FoxO3a, ERK, β-catenin, and ROS) or cell-membrane receptors for communication with the microenvironment (CXCR4, CXCR7, VLA-4, and TLR). Eleven nodes conform the MSC subsystem, integrated by intracellular signaling molecules (PI3K/Akt, NF-κB, GSK3β, FoxO3a, ERK, β-catenin, and ROS), a gap-junction protein regulating communication among MSC (Cx43), communication ligands with HSPC (VCAM-1 and CXCL12) and TLR. Common internal nodes in both HSPC and MSC systems are representative molecules from the most studied pathways influencing proliferation, migration, survival, and -some of them- differentiation. Finally, the microenvironmental compartment is represented by G-CSF secreted by myeloid and stromal cells (Majumdar et al., 2000; Allakhverdi et al., 2013; Tesio et al., 2013; Boettcher et al., 2014), its inductor IL-1 which is secreted by MSC and HSPC, and lTLR so as to model a homeostasis disruption that is known to drive a pro-inflammatory signaling. Model inputs are Cx43 and lTLR, while the activation value of the other 24 nodes is dependent on the network topology and the initial state of the input nodes. All logical rules used for the computational simulation with BoolNet are shown in Table 1. Note that the logical rules for the input nodes include self-regulations, but these are for computational purposes to represent their sustained activation, rather than a biological reality.

**Table 1:** **Logical rules used for HSPC-MSC modeling as a Boolean system on BoolNet**.

**Node**	**Logical rule**
Bcatenin_H	!GSK3B_H
CXCR4_H	CXCL12_M & !(CXCR7_H | GCSF | Gfi1_H)
CXCR7_H	CXCL12_M & NfkB_H
ERK_H	((CXCR4_H & PI3KAkt_H) | CXCR7_H | GCSF | Gfi1_H | ROS_H | VLA4_H ) & !(FoxO3a_H | GSK3B_H)
FoxO3a_H	(Bcatenin_H | ROS_H) & !(ERK_H | PI3KAkt_H)
Gfi1_H	(GCSF | TLR_H) & !Gfi1_H
GSK3B_H	!PI3KAkt_H
NfkB_H	(TLR_H | ROS_H | (IL1 & PI3KAkt_H)) & !(FoxO3a_H)
PI3KAkt_H	((CXCR4_H & CXCR7_H) | GCSF | ROS_H | TLR_H | VLA4_H) & !FoxO3a_H
ROS_H	IL1 & TLR_H & (!FoxO3a_H)
TLR_H	lTLR
VLA4_H	VCAM1_M & CXCR4_H
Cx43_M	Cx43_M
Bcatenin_M	!(FoxO3a_M | GSK3B_M | NfkB_M)
CXCL12_M	Cx43_M & !(Bcatenin_M | GCSF | NfkB_M)
ERK_M	GCSF | ROS_M | TLR_M
FoxO3a_M	(Bcatenin_M | ROS_M) & !(ERK_M | PI3KAkt_M)
GSK3B_M	!PI3KAkt_M
NfkB_M	(IL1 & PI3KAkt_M) | (ROS_M & ERK_M) | TLR_M
ROS_M	IL1 & TLR_M & (!FoxO3a_M)
PI3KAkt_M	GCSF | ROS_M | TLR_M
TLR_M	lTLR
VCAM1_M	!Bcatenin_M | NfkB_M | PI3KAkt_M
lTLR	lTLR
IL1	((ROS_M | NfkB_M) & !PI3KAkt_M) | ((ROS_H | NfkB_H) & !PI3KAkt_H)
GCSF	IL1

*Nodes representing molecules in HSPC are denoted with “_H” at the end of the node name, while nodes representing molecules in MSC are denoted with “_M.” Logical rules were constructed using the logical operators AND (&), OR ( | ) and NOT ( ! ). The corresponding common names and genes ID are found in Table S3*.

### Attractors of the wild-type network: searching for the relevance of TLR in the biology of CXCL12

**Figure 2:**
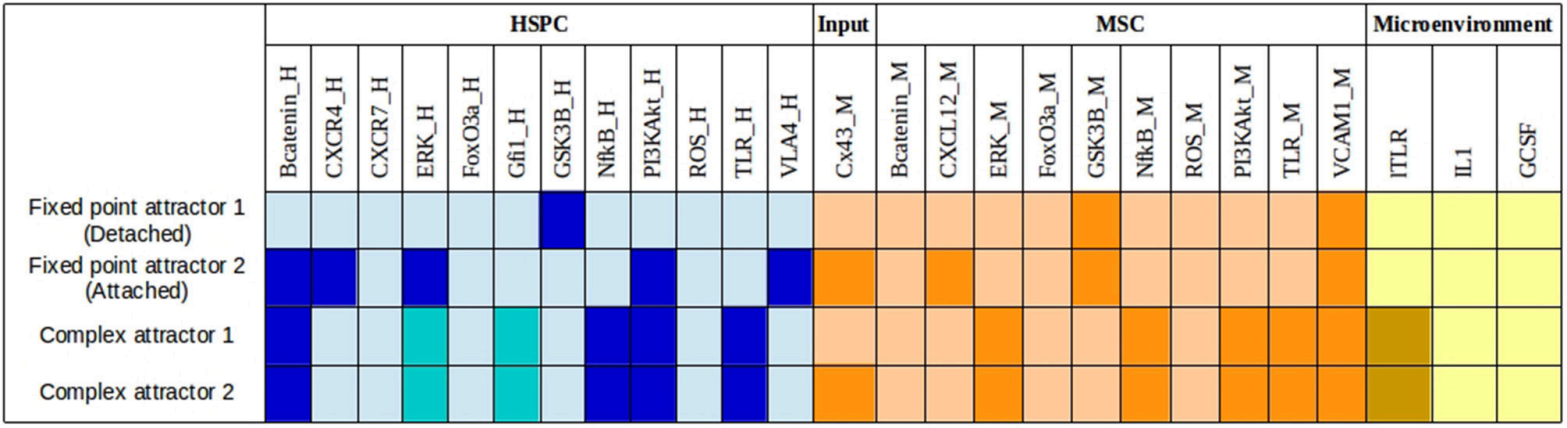
**Asynchronous attractors from the wild type network**. Dark color denotes an activation value of 1, while light color represents an activation value = 0. The blue, orange, and yellow colors distinguish the nodes in the three compartments in the HSPC-MSC network corresponding to HSPC, MSC, and microenvironmental factors, respectively. The last two attractors obtained when the initial states for the asynchronous simulation had lTLR value = 1, have two nodes (ERK_H and Gfi1_H) whose activation values oscillate and are responsible of the complex attractor. Oscillatory values are represented by intermediate blue color. Nodes representing molecules in HSPC are denoted with “_H” at the end of the node name, while nodes representing molecules in MSC are denoted with “_M.”

The asynchronous simulation of the Boolean model returned 4 attractors: 2 fixed points and 2 complex attractors (Figure 2). The first two attractors, fixed point attractor 1 and 2, were identified with the physiological detached and attached state of the HSPC with its MSC counterpart, respectively.

Both fixed point attractors will depend on the initial states of both, TLR and Cx43. Thus, in the absence of lTLR, the final fates will depend on the initial activation state of Cx43. However, once TLR is activated, final fates are not contributed anymore from the activation state of Cx43.

Loss of HSPC-MSC communication corresponding to a detachment state, is due to the absence of Cx43 and the consequent inactivation of CXCL12. In the activation pattern of this attractor, only VCAM-1 accompanied by GSK3β in both sub systems remained active (Tabe et al., 2007). On the contrary, when Cx43 is active (as in fixed point attractor 2), CXL12 is expressed by the MSC, which in turn positively regulates the CXCR4 receptor required for the activation of the VLA-4/VCAM-1 axis. The pattern in HSPC, correspond to ERK and PI3K/Akt activation, well-described elements downstream CXCR4 and VLA-4 (Tabe et al., 2007). β-catenin, a subject of debate about its function on stem cell maintenance, is turned on as a consequence of the GSK3β inhibition by PI3K/Akt (Dao et al., 2007).

**Figure 3:**
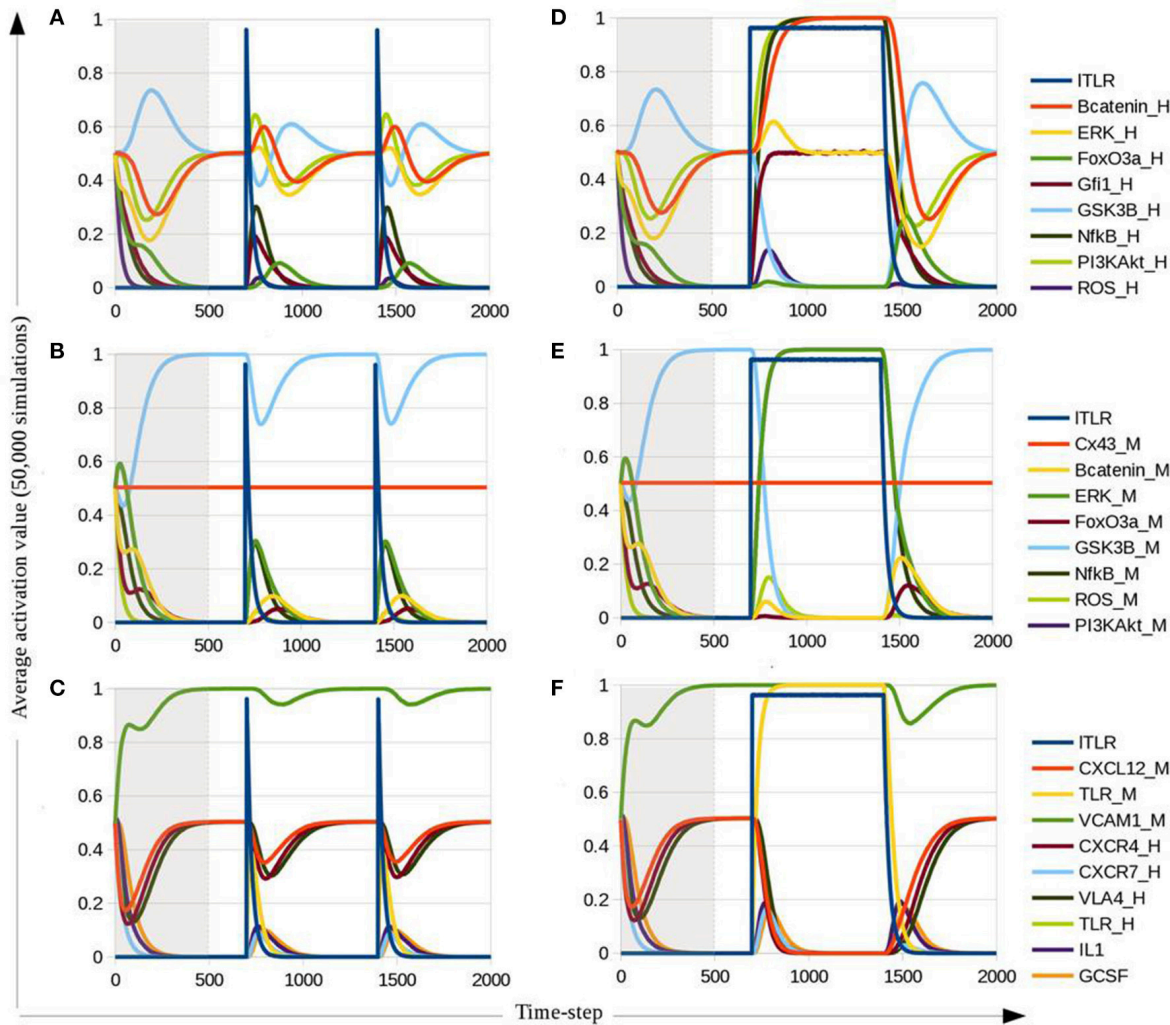
**Average activation value for intracellular HSPC nodes (A,D), intracellular MSC nodes (B,E) and communication axes among HSPC, MSC, and microenvironment (C,F). (A–C)** Correspond to simulations with a short (1 time-step) stimulation of lTLR at time-steps 700 and 1400. **(D–F)** Correspond to simulations with lTLR stimulation at time-step 700 with a length of 699 time-steps. Nodes representing molecules in HSPC are denoted with “_H” at the end of the node name, and nodes representing molecules in MSC are denoted with “_M.” Gray area covers the stabilization time steps until attractors are reached.

Complex attractors 1 and 2 share the same activation values in all nodes, except for the initial state of Cx43 which is an input and therefore may be consistently either active or inactive through simulation. Importantly, these two attractors have the node for ITLR active, so that under induced pro-inflammatory conditions the resultant perturbation of CXCR4/CXCL12 and VLA-4/VCAM-1 is exclusively dependent on CXCL12 down regulation in MSC by NF-κB. The network attractors are concordant with experimental observations (Ueda et al., 2004; Wang et al., 2012; Yi et al., 2012) with the exception of IL1 and GCSF inactivation although lTLR-induced NF-κB signaling in hematopoietic and mesenchymal compartments. In order to explain this discrepancy we may remark that an attractor is a stable network state or set of states, reached after the network went through a sequence of transient states where, in most biological systems, there is cross-pathway communication for modulating cellular response (Williams et al., 2004; Tapia-Abellán et al., 2014), so IL1 and GCSF could be activated in some transient states but down-regulated by other pathways responding to lTLR activation. Due to the existence of regulatory circuits among pathways, in the presence of ITLR there is an oscillatory behavior of ERK and Gfi1. Therefore, we applied the dynamic multicellular approach described by Wu et al. (2009) in order to have a deeper understanding of the HSPC-MSC model upon perturbations. The average activation value of 50,000 simulations for all nodes within the HSPC-MSC network was plotted and presented in Figure 3. The plots represent a qualitative approach for the analysis of the cell population trend under specific conditions. Considering that the initial activation values are randomly chosen, with exception of lTLR, TLR_M, and TLR_H which activation value was set to 0, the average initial activation value for the rest of the nodes correspond to 0.5. From time-step 0 to time-step 499 correspond to the stabilization of the dynamics. Of note, the plateau obtained around time-steps 500-699 corresponds to the average of the two fixed point attractors.

### Analysis of transitory states applied to a multicellular approach: from pro-inflammatory signals to CXCL12 downregulation

The short lTLR stimulation at time-step 700 and 1400 (Figures 3A–C) induces up-regulation of Gfi1 in HSPC (Figure 3A), and of NF-κB and PI3K/Akt in both HSPC and MSC compartments (Figures 3A,B). These nodes maintain a sustained activation as long as the lTLR is present (Figures 3D–F). In contrast, ERK, ROS and FoxO3a showed an increase but are regulated by other nodes, providing a feedback to basal values. Accompanying the cross-regulation of intracellular pathways, a decrease on CXCR4, CXCL12, VLA-4, and VCAM-1 activation is observed. As expected, there is positive signaling of the pro-inflammatory cytokines with a parallel co-increase of CXCR7, signals damped by PI3K/Akt and CXCL12 down-regulation, respectively.

### Model validation by mutant analysis

Listed in Table 2 are the observations from comparisons between the resultant attractors of simulations with null (“*loss of function”*) and constitutive expression mutants (“*gain of function”*), against the wild-type model. We focused on the activation value changes in the two axes of interest –CXCR4/CXCL12 and VCAM-1/VLA-4. Even though the nodes included in the reconstruction of the present model are well-studied elements of cell fate related-pathways, there is a lack of experiments correlating their perturbation with microenvironment modifications that impact HSPC behavior (Table 2, Table S4). Due to this missing data, and in order to validate the model, we now used available information of general alterations in hematopoiesis in the presence of lTLR.

**Table 2:** **Results from the model outcome for single node mutations**.

**Loss of function**		
**Node**	**Model outcome**	**Experimental evidence**
Bcatenin_H, CXCR7_H, ERK_H, FoxO3a_H, NfkB_H, ROS_H, Bcatenin_M, ERK_M, FoxO3a_M, NfkB_M, ROS_M, IL1, GCSF	No changes in the CXCR4/CXCL12 and VLA-4/VCAM-1 axes with respect to the attractors from the wild-type model.	Cobas et al., 2004; Jeannet et al., 2008; Sierro et al., 2007
CXCL12_M, CXCR4_H	Loss of CXCR4/CXCL12 and VLA-4/VCAM-1 in the fixed point attractor with active Cx43_M.	Greenbaum et al., 2013; Sugiyama et al., 2006; Tzeng et al., 2011
Gfi1_H	No changes in the CXCR4/CXCL12 and VLA-4/VCAM-1 axes. Stabilization of lTLR-dependent complex attractors with no activation of ERK_H .	Hock et al., 2004; Zeng et al., 2004
GSK3B_H	Additional fixed point attractor when Cx43 is active, where FoxO3a_H is up-regulated and repressing PI3K_H and ERK_H. Also, are additional complex attractor in the presence of lTLR where FoxO3a_H inhibits PI3KAkt_H, ERK_H and NfkB_H activation.	Holmes et al., 2008
PI3KAkt_H, PI3KAkt_M	No changes in CXCR4/CXCL12 and VLA-4/VCAM-1 axes with respect to the attractors from the wild-type model. Under lTLR stimulation, pro-inflammatory cytokines turned on and in consequence ROS_H. In PI3KAkt_H null mutant, ERK_H is inhibited in every condition and FoxO3a_H is intermittently activated under lTLR stimulation.	Williams et al., 2004; Champelovier et al., 2008; Xu et al., 2012
VLA-4, VCAM1_M	PI3KAkt_H and ERK_H are turned off even if CXCR4/CXCL12 axis is active.	Wang et al., 1998; Scott et al., 2003
GSK3B_M	Fixed point attractors are lost and became complex attractors. Activation of Cx43, leads to two complex attractors of which one activates CXCR4/CXCL12 and VLA-4/VCAM-1 axes intermittently. In the absence of Cx43, two complex attractors are generated, and one of them unsteadily activate IL1 and GCSF.	Satija et al., 2013
**Gain of function**		
**Node**	**Model outcome**	
GSK3B_M, ERK_M, VCAM1_M, FoxO3a_M	No changes in the CXCR4/CXCL12 and VLA-4/VCAM-1 axes with respect to the attractors from the wild-type model.	NE (Not experimental evidence found)
CXCR7_H, NfkB_H, Bcatenin_M, NfkB_M, PI3KAkt_M, GCSF, IL1	Loss of CXCR4/CXCL12 and VLA-4/VCAM-1 in the fixed point attractor with active Cx43_M.	Cortez et al., 2013; Kode et al., 2014
Bcatenin_H	Under the activation of Cx43_M, an alternative steady state is reached where PI3KAkt_H and ERK_H are not expressed and instead, FoxO3a_H and GSK3B_H are active despite the activation of CXCR4_H and VLA4_H.	Kirstetter et al., 2006; Champelovier et al., 2008
CXCL12_M	Under lTLR stimulation, the complex attractors show a sustained activation of CXCR7_H.	NE
FoxO3a_H	Bcatenin_H, ERK_H and PI3KAkt_H inactivation under any condition.	Yamazaki et al., 2006
Gfi1_H	Loss of CXCR4/CXCL12 and VLA-4/VCAM-1 in the fixed point attractor with active Cx43_M. Stabilization of lTLR-dependent complex attractors.	Hock et al., 2004; Khandanpour et al., 2013
GSK3B_H	Inhibition of ERK_H and Bcatenin_H when CXCR4_H or lTLR are active.	NE
PI3KAkt_H	Bcatenin_H remains active in the absence of Cx43 and lTLR.	Wang et al., 2013
ROS_H, ROS_M	Loss of CXCR4/CXCL12 and VLA-4/VCAM-1 in the fixed point attractor with active Cx43_M. ROS_M overexpression mutant, activates PI3K_M, which in consequence inhibits FoxO3a_M.	Lu et al., 2013; Zhang et al., 2015
VLA-4	Constitutive activation of PI3KAkt_H, ERK_H and Bcatenin_H.	Schofield et al., 1998; Shalapour et al., 2011

MSC ERK, FoxO3a, and PI3K/Akt nodes participating in CXCR4/CXCL12 and VCAM-1 VLA-4 axes regulation were not found in the revised literature. β-catenin in MSC has a role on osteoblastogenesis and its constitutive induced expression in osteoblasts in a mice model results in acute myeloid leukemia (AML) induction (Kode et al., 2014). The constitutive expression of β-catenin showed an outcome where, under non-induced inflammation, the CXCR4/CXCL12 axis is disrupted. This gives support to our hypothesis that CXCR4/CXCL12 is probably involved in the maintenance of leukemic cells. Furthermore, the dynamic multicellular approach in the gain of function of β-catenin in MSC, reproduced the recovery of VCAM-1 expression upon stimulation of lTLR as reported by Kincade in OP9 cells (Figure S1; Malhotra and Kincade, 2009).

GSK3β inhibition in MSC has been known to function in the regulation of osteoblast and adipocyte differentiation. Besides, experimental effect of a GSK3β-inhibitor on osteoblastogenesis has shown that the decrease of this kinase induces down-regulation of CXCL12 expression (Satija et al., 2013). The model is consistent with the unsteadiness of CXCL12 activation in the simulation of the mutant (Figures S2A,B).

According to our hypothesis, a pro-inflammatory-induced CXCR4/CXCL12 disruption results in leukemic progression support. In the proposed model, overexpression of NF-κB disrupts the HSPC-MSC communication (Figure S2C). This is in agreement with the reported leukocytosis associated to up-regulation of NF-κB within BM MSCs from a mice model of high-fat diet (Cortez et al., 2013). Finally, modeling of a gain of function mutation in ROS resulted in the blocking of CXCL12 activation (Figure S2D). This is also in accordance of the recent report of oxidative damage induced by iron in MSC, resulting in down-regulation of CXCL12 expression and reduction of their hematopoietic supporting function (Zhang et al., 2015). Moreover, the iron-induced hematopoietic alterations previously observed by other groups, are attenuated by the treatment with ROS inhibitors (Lu et al., 2013).

Nodes in HSPC which have been experimentally reported as dispensable for hematopoiesis, which did not show any alterations in the CXCR4/CXCL12 and VLA-4/VCAM-1 axes on the mutant simulations, are β-catenin (Figures S3A–D; Cobas et al., 2004; Jeannet et al., 2008) and CXCR7 (Figures S3E,F). However, even though *in vivo* β-catenin null mutant HSPC does not lose long-term reconstitution capacity or multipotentiallity, its overexpression produces lose of stemness and differentiation blockage to erythroid and lymphoid lineages (Kirstetter et al., 2006; Scheller et al., 2006). Simulations of the gain of function of β-catenin resulted in the appearance of additional attractors where FoxO3a and GSK3β are increased (Figures S4A,B, S5B), suggesting a reduction in proliferation and/or apoptosis induction (Maurer et al., 2006; Yamazaki et al., 2006). In turn, the simulation of overexpression of FoxO3a showed a down-regulation of ERK and PI3K (Figures S4C, S5C). Also reported as proliferative repressors in HSPC (Hock et al., 2004; Zeng et al., 2004; Holmes et al., 2008), Gfi1 and GSK3β overexpression mutants inhibited ERK activation, and additionally Gfi1 induce the downregulation of PI3K/Akt node, CXCR4/CXCL12 and VLA-4/VCAM-1 axes (Figures S4 and S5). Disagreeing with experimental data (Holmes et al., 2008), GSK3β null mutant outcome result in an additional attractor where PI3K/Akt and ERK are inactive, notwithstanding CXCR4 and VLA4 activation (Figure S6).

**Figure 4:**
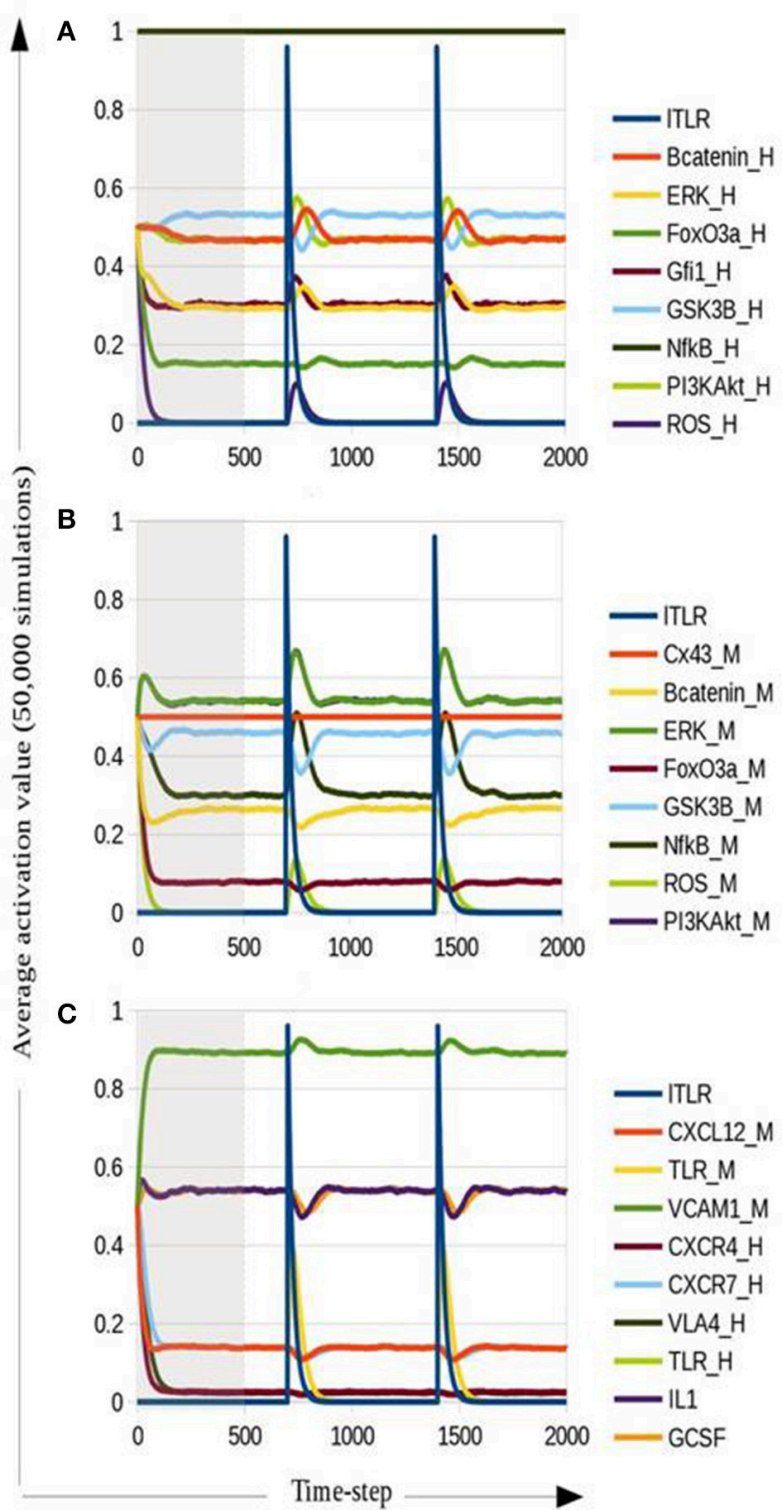
**Dynamic multicellular simulation for a ALL simplified model addressed by NF-κB gain of function in HSPC**. Average activation for intracellular HSPC nodes **(A)**, intracellular MSC nodes **(B)** and communication axes among HSPC, MSC, and microenvironment **(C)**. Nodes representing molecules in HSPC are denoted with “_H” at the end of the node name, while nodes representing molecules in MSC are denoted with “_M.” Gray area covers the stabilization time steps until attractors are reached.

Of interest, NF-κB (Figure 4) and ROS (Figures S4F, S5F) constitutive expression in HSPC induce additional attractors with activation of IL-1 and G-CSF, and inhibition of axes regulating HSPC-MSC contact. A number of investigations on cancer cells report a correlation of NF-κB increased levels and CXCR4 (Richmond, 2002; Ayala et al., 2009; Shin et al., 2014). Nonetheless, a recent study in human leukemic cell lines has shown that LPS treatment increases MMP-9 activity, a metalloproteinase known to efficiently degrade CXCR4 and CXCL12 (Hajighasemi and Gheini, 2015).

### NF-κB gain of function mutant as ALL simplified model

How common alterations in ALL cells may induce BM microenvironment remodeling, regardless of the underlying genetic aberration, was investigated by running a dynamic multicellular simulation using the mutant network for NF-κB gain of function within the HSPC sub-system. The results shown in Figure 4 confirm that NF-κB mutation in HSPC may perturb HSPC-MSC communication in parallel with the induction of other alterations previously reported in ALL cells, such as the increase of Gfi1 expression (Purizaca et al., 2013) and a pro-inflammatory milieu (Vilchis-Ordoñez et al., 2015). IL1 and G-CSF activation by HSPC up-regulate ERK, NF-κB and PI3K/Akt in MSC. As consequence of PI3K/Akt increase in MSC, β-catenin is up-regulated through the inhibition of GSK3β. Strikingly, the sustained activation of CXCR7 resulted as a consequence of NF-κB constitutive expression in HSPC and CXCL12 residual expression from MSC. CXCR7/CXCL12 axis was recently reported to be increased in ALL cells and a possible participation in abnormal cell migration was suggested (Melo et al., 2014).

## Discussion

According to the classical model of hematopoiesis, normal blood cells are replenished throughout life by stem and early progenitor populations undergoing stepwise differentiation processes in the context of intersinusoidal specialized niches (Purizaca et al., 2012; Vadillo et al., 2013). Cell cycle status, self-renewing capability and the central cell fate decisions depend, in great part, on the microanatomic organization and signals from the BM environment. Endosteal, perivascular and reticular niches provide support by cell-cell interactions and growth/differentiation factors that control the expression of lineage-specific transcription factors, among other elements. Within the reticular niche, mainly composed by CXCL12-abundant reticular cells (CARs), a special category of MSCs, the chemokine CXCL12 and its receptor CXCR4 play a pivotal role in the regulation of lymphopoiesis from the earliest stages of the pathway (Tokoyoda et al., 2004; Nagasawa, 2015). The transcription factor Foxc1 governs CXCL12 and stem cell factor expression, allowing the CAR niche formation for maintenance of HSC, common lymphoid progenitors, B cells, NK and plasmacytoid dendritic cells (Omatsu et al., 2014). The net balance of its disruption is instability of adaptive and innate immune cell production. Recent findings suggest that elevation of cytokines and growth factors, including G-CSF and TNFα, due to infectious stress, substantially reduce the expression of CXCL12, SCF and VCAM-1, further impairing primitive cell maintenance and prompting their proliferation and migration (Kobayashi et al., 2015, 2016).

Much remains to be unraveled about CXCL12-related mechanisms of intercommunication damage that may favor growth of cancer cells at the expense of healthy hematopoiesis during biological contingencies such as hematological malignancies and biological stress. Although, genetic heterogeneity may be co-responsible for differences in ALL overall survival, response to treatment, differentiation-stage arrest or even predisposition to metastasis, a common need might be the development of biological features that provide pre-malignant cells decisive advantage over normal cells to compete for the same ecological niche. Given the importance of CXCR4/CXCL12 axis for homeostatic hematopoiesis and of its presumptive disruption in ALL BM, we now propose a Boolean model reconstructed with some of the most studied elements upstream and downstream this key communication axis. Our model shows its capacity to simulate several phenotypes relevant to ALL. According to previous experimental research, the major assumption made from this model is that the integrity of CXCR4/CXCL12 signaling, promoting the required activation of the VLA-4/VCAM-1 integrins interaction, is absolutely necessary for HSPC retention in the mesenchymal niche and in consequence, indispensable for optimal hematopoiesis regulation (Lévesque et al., 2003; Lua et al., 2012; Greenbaum et al., 2013; Park et al., 2013). The HSPC-MSC model asynchronous simulation in the absence of lTLR returned two attractors corresponding to HSPC attachment and detachment to MSC. The ‘attachment’ status, represented by the induction of CXCR4/CXCL12 and/or VLA-4/VCAM-1 axes, also exhibited PI3K/Akt and β-catenin activation within the HSPC compartment. Although there is some controversy about the β-catenin role in HSC regulation (Kirstetter et al., 2006; Duinhouwer et al., 2015), the co-activation of PI3K/Akt and β-catenin is known to promote self-renewal and HSC expansion (Perry et al., 2011). Two core pathways downstream CXCR4/CXCL12 binding are PI3K/Akt and ERK, both promoters of cell survival and regulators of proliferation. Considering that the mesenchymal stromal niche has being identified as the interface between the quiescence promoting osteoblastic niche and the vascular niche regulating final lineage commitment and cell migration, the signals provided by mesenchymal cells should tightly regulate proliferation/expansion in order to further allow differentiation. According to this statement, the attractor representing the detached state conducts to pro-apoptosis signaling in the absence of aberrant expression of NF-κB, that relies on cytochrome C release-associated normal functions of GSK3β in HSPC (Maurer et al., 2006).

By using elegant mice disease models and controlled culture systems, a wealth body of studies has recently highlighted the co-participation of inflammation and infectious stress in the HSPC exit from quiescence status, as well as in cancer etiology and progression (Baldridge et al., 2011; Vilchis-Ordoñez et al., 2015). Chronic inflammation and carcinogenesis have been closely connected via either a oncogenes-derived intrinsic pathway or through an extrinsic pathway from external factors that promote latent inflammatory responses involving signaling pathways such as MyD88, NF-κB, and STAT3 (Mantovani et al., 2008; Krawczyk et al., 2014).

Interestingly, pattern recognition receptors (PRRs), including Toll-like receptors (TLRs) are functionally expressed from the most primitive stages of hematopoiesis and contribute to emergent cell replenishment in response to life-threatening infections or disease-associated cell damage (Nagai et al., 2006; Welner et al., 2008; Dorantes-Acosta et al., 2013; Vadillo et al., 2014). This phenomenon is called emergency hematopoiesis and is regulated at the most primitive cell level (Kobayashi et al., 2015, 2016).

The potential relevance of this mechanism in leukemogenesis was the focus of this investigation, and our model allowed for the analysis of most behaviors observed under experimental settings. The discrete simulation of NF-κB constitutive expression mutant on HSPC, gave further support to our hypothesis on the perturbation of CXCR4/CXCL12 communication axis induced by pro-inflammatory microenvironment. The single mutation of NF-κB was sufficient to remodel the dynamical behavior of the three sub-systems represented, which was an unexpected behavior of the model. The dynamic analysis of the ALL-like network, also suggested the activation of an alternative communication pathway mediated by CXCR7 binding CXCL12. Inhibition of CXCL12 within the mesenchymal niche, may be fundamental for cell migration to adjacent BM structures unable to sustain proper differentiation or even to extramedullar tissues, accounting for a predictable role of this axis in metastasis.

## Concluding remarks

The proposed HSPC-MSC model is the first systemic approximation to understand the intercommunication pathways underlying primitive cell retention/proliferation in the mesenchymal niche as a determinant factor for progression of hematological hyperproliferative diseases. We applied conventional discrete dynamical modeling and non-conventional population-like approaches as an average behavior of the network model. Future improvement of discrete dynamical modeling for ALL system will provide a powerful tool for investigation of unbalanced competitions between leukemic and normal hematopoietic cells within the BM. Overall, systems biology will advance our comprehensive view of the mechanisms involved in the pathogenesis of leukemic niches that may illuminate therapeutic strategies based on cell-to-cell crosstalk manipulation.

## Author contributions

JE designed the work; generated, analyzed and interpreted data; wrote the paper. HM interpreted data; revised the work for intellectual content; wrote the paper. LM designed the work; interpreted data; revised the work for intellectual content; wrote the paper. RP designed the work; interpreted data; revised the work for intellectual content; wrote the paper.

### Conflict of interest statement

The authors declare that the research was conducted in the absence of any commercial or financial relationships that could be construed as a potential conflict of interest.
