# Supplementary material for: Modeling the Pro-inflammatory Tumor Microenvironment in Acute Lymphoblastic Leukemia Predicts a Breakdown of Hematopoietic-Mesenchymal Communication Networks
Source: Front Physiol. 2016 Aug 19;7:349. doi: 10.3389/fphys.2016.00349 (PMC4990565; doi:10.3389/fphys.2016.00349)
Supplement: Supplementary file 1 [file Table1.doc]

**SUPPLEMENTARY INFORMATION**

Table S1. Public experimental information used for the establishment of the logical rules for the HSPC sub-system in the HSPC-MSC model.

| **Input node** | **Type of interaction** | **Target node** | **Bibliographic report** | **Reference** | **Experimental model (H, human; M, mice; R, rat)** |
| --- | --- | --- | --- | --- | --- |
| GSK3β (GSK3B_H) | -| | β-catenin (Bcatenin_H) | GSK3β-mediated phosphorylation of β-catenin, triggers its ubiquitination and subsequent proteasome-dependent proteolysis. | (Perry et al., 2011)⁠ | HSC (M) |
| CXCL12 (CXCL12_M) | -> | CXCR4 (CXCR4_H) | CXCL12 mainly binds to the membranal G-protein-coupled receptor, CXCR4. CXCR4 plays an important role in HSPC maintenance in the bone marrow, besides its participation in the regulation of proliferation and quiescence. | (Peled et al., 2000; Spiegel et al., 2004; Sugiyama et al., 2006)⁠ | HSPC (H), Pre-B ALL cells (H) and HSC (M). |
| CXCR7 (CXCR7_H) | -| | CXCR4 (CXCR4_H) | In cells expressing both known CXCL12 receptors, CXCR4 and CXCR7, CXCR7 show a higher affinity to CXCL12 and β-arrestin, inhibiting the G-protein signaling by CXCR4. Furthermore, CXCR7 heterodimerize with CXCR4 promoting its internalization and degradation. | (Coggins et al., 2014; Melo et al., 2014; Torossian et al., 2014; Uto-Konomi et al., 2013)⁠ | T-ALL cells (H), Jurkat T cells (H), breast cancer cells (H), HSPC (H) and in silico analysis. |
| G-CSF (GCSF) | -| | CXCR4 (CXCR4_H) | Increased G-CSF secretion, up-regulates neutrophil elastase and cathepsin-G expression in the bone marrow. Both proteases, cleave CXCR4 in the middle site between the first and the second transmembranal domains inhibiting its chemotactic property. | (Lévesque et al., 2003)⁠ | B-ALL cell line (H) and HPC (H). |
| Gfi1 (Gfi1_H) | -| | CXCR4 (CXCR4_H) | In myeloid cells has been reported the repressor activity of Gfi1 transcriptional factor on CXCR4 expression, due to its direct binding to the promoter region of CXCR4 gene*.* On the other hand, HSC collected from Gfi1b^ko/ko^ mice have shown an elevated expression of CXCR4. | (Khandanpour et al., 2010; De La Luz Sierra et al., 2007)⁠ | Myeloid cell line (M) and HSC (M). |
| CXCL12 (CXCL12_M) | -> | CXCR7 (CXCR7_H) | CXCR7 is an atypical chemokine receptor whose signiling is mediated by β-arrestin and not by G-protein. CXCR7 is an alternative CXCL12 receptor that has shown a higher affinity for CXCL12 than CXCR4, however, it has a lower expression on HPC. | (Burns et al., 2006; Tarnowski et al., 2010; Torossian et al., 2014)⁠ | Endothelial cell line (H), breast cancer (H) and hematopoietic cells (H), peripheral blood-derived mononuclear cells (H) and HSPC (H). |
| NF-κB (NfkB_H) | -> | CXCR7 (CXCR7_H) | CXCR7 gene promoter contains three consensus sites for NF-κB binding, from which only two of them proved to play a crutial role in CXCR7 transcription regulation. | (Tarnowski et al., 2010)⁠ | Hematopoyetic cell lines (H) |
| PI3K -> PIP3 -> Akt AND CXCR4 (PI3KAkt_H & CXCR4_H) | -> | ERK (ERK_H) | Induced ERK1/2 phosporylation after CXCL12 binding to CXCR4 in dendritic cells, is downregulated with PI3K-inhibitor treatment. | (Delgado-Martín et al., 2011)⁠ | Dendritic cells (H) |
| CXCR7 (CXCR7_H) | -> | ERK (ERK_H) | Under CXCR4 blocking conditions, CXCR4 binding to CXCR7 promotes ERK1/2 phosphorylation and activation. | (Kumar et al., 2012) | Jurkat T cells (H) |
| FoxO3a (FoxO3a_H) | -| | ERK (ERK_H) | FoxO3a positively regulates Spred2 expression, which in turn inhibits ERK phosphorylation. | (Miyamoto et al., 2008)⁠ | HPC (M) |
| G-CSF (GCSF) | -> | ERK (ERK_H) | G-CSF treatment, promote an increase in phosphorylation levels of Akt and ERK1/2. | (Furmento et al., 2014; Liu et al., 2007)⁠ | Trophoblast cell line (H) and HPC (M). |
| GSK3β (GSK3B_H) | -| | ERK (ERK_H) | GSK3β inhibits LPS-induced ERK1/2 phosphorylation. Inhibition of GSK3β promotes a hyperactivation of ERK1/2. | (Noh et al., 2012)⁠ | Dendritic cells (M) |
| Gfi1 (Gfi1_H) | -> | ERK (ERK_H) | Mononuclear cells from Gfi1^-/-^  mice stimulated with G-CSF, showed a decreased activation of ERK pathway. | (De la Luz Sierra et al., 2010)⁠ | Bone marrow-derived mononuclear cells (M) |
| ROS (ROS_H) | -> | ERK (ERK_H) | Oxidative stress promotes MAP kinases activation increasing ERK1/2 phosphorylation. | (Griffith et al., 1998; Keshari et al., 2013)⁠ | Neutrophils (M) and Jurkat T cells (H). |
| VLA-4 (VLA4_H) | -> | ERK (ERK_H) | The interaction between VLA-4 integrin with its ligands, activates the integrin linked kinase which in turn, up-regulates ERK pathway activation. | (McGilvray et al., 1997; Tabe et al., 2007)⁠ | AML cell line (H) |
| β-catenin (Bcatenin_H) | -> | FoxO3a (FoxO3a_H) | β-catenin interacts directly with FoxO1, FoxO3a and FoxO3, augmenting its transcription activity. | (Essers et al., 2005)⁠ | B-cell Lymphoma cells (H), adenocarcinoma cells (H) and yeast two-hybrid system. |
| ERK (ERK_H) | -| | FoxO3a (FoxO3a_H) | ERK phosphorylates serine 294, 344 and 425 of FoxO3a causing its inactivation and promoting its degradation via MDM2. | (Yang et al., 2008)⁠ | Breast cancer cells (H), hepatoma cells (H) and embrionic fibroblasts (M). |
| PI3K -> PIP3 -> Akt (PI3KAkt_H) | -| | FoxO3a (FoxO3a_H) | Akt phosphorylate threonine 32, serine 253 and serine 315 of FoxO3a promoting its cytoplasmic localization and down-regulating its target genes. | (Brunet et al., 1999; Miyamoto et al., 2008; Park et al., 2008)⁠ | HSC (Miyamoto, M) |
| ROS (ROS_H) | -> | FoxO3a (FoxO3a_H) | Accumulation of ROS promote the nuclear localization of FOXO, including FoxO3a, increasing the transcription of ROS-regulating enzymes, such as catalase. Other suggested mechanisms for ROS-mediated FOXO transcriptional activation, involve JNK pathway, acetylases and GTPase Ral activity. | (Brunet et al., 2004; Essers et al., 2005)⁠ | Embrionic kidney cells (H), colon carcinoma cells (H), embrionic fibroblasts (M) and myoblastos (M). |
| G-CSF (GCSF) | -> | Gfi1 (Gfi1_H) | Gfi1 mRNA concentration is increased in mononuclear cells treated with G-CSF. This stimulation is dependent on the integrity of the carboxil terminal region of the G-CSF receptor. | (De La Luz Sierra et al., 2007; de la Luz Sierra et al., 2010; Zhuang et al., 2006)⁠ | Bone marrow-derived cell suspesions (M) and myeloid cell line (M). |
| Gfi1 (Gfi1_H) | -| | Gfi1 (Gfi1_H) | There have been identified consensus sites for Gfi1 binding in its own promoter region, suggesting a *cis* regulation. This sites are apparently conserved in mice, rats and humans. | (Doan et al., 2004; Yücel et al., 2004)⁠ | Jurkat T cells (H), and B and T lymphocytes (M). |
| TLR4 (TLR_H) | -> | Gfi1 (Gfi1_H) | Transcriptional repressor Gfi1 is upregulated after LPS stimulation of bone marrow macrophages. Gfi1 binds to p65, regulating the induction of some NF-κB classical targets. | (Sharif-Askari et al., 2010)⁠ | Macrophages (M), AML-derived monocytes (H) and embrionic fibroblasts (M). |
| PI3K -> PIP3 -> Akt (PI3KAkt_H) | -| | GSK3β (GSK3B_H) | Akt activation phosphorylates and inactivate GSK3β. | (Tjin et al., 2006)⁠ | B-cell lymphoma (M) and diffuse large B-cell lymphoma cells (H). |
| NF-κB AND NOT Gfi1 (NfkB_H & !Gfi1_H) | -> | IL-1β (IL1) | From the analysis of IL-1β promoter region in rainbow trout, has been observed the existence of consensus sites for NF-κB, NF-IL6, AP1, AP4, CHOP/CEBPa, SP1, PU.1 y Gfi1. NF-κB-binding sites conservation in human and mice has been confirmed through *in silico* analysis. Gfi1 has also been reported to regulate the activation of IL-1β by NF-κB upon TLR-4 activation. | (Jones et al., 2001; Sharif-Askari et al., 2010; Tak and Firestein, 2001; Wang et al., 2002)⁠ | Fibroblasts (chinese hamster), macrophages (M) and *in silico* analysis (H, M) |
| PI3K -> PIP3 -> Akt (PI3KAkt_H) | -| | IL-1β (IL1) | PI3K/Akt pathway is involved in the regulation of IL-1β maturation due to the inhibition of caspase-1 required for IL-1β maturation. PI3K/Akt has been also associated to the up-regulation of antagonist molecules for the IL-1β receptor, regulating also IL-1β signaling. | (Molnarfi et al., 2006; Tapia-Abellán et al., 2014)⁠ | Monocytes (H) and macrophages (H). |
| ROS (ROS_H) | -> | IL-1β (IL1) | Macrophages *in vitro* stimulation with LPS, upregulates pro-IL-1β transcription and IL-1β exportation though ROS-dependent signaling. | (Gabelloni et al., 2013; Hsu and Wen, 2002) | Neutrophils (H) and macrophages (M). |
| FoxO3a (FoxO3a_H) | -| | NF-κB (NfkB_H) | FoxO3a inhibits the traslocation of the NF-κB subunit, RelA. | (Lin et al., 2004)⁠ | Epithelial embrionic kidney cells (H), B-cell lymphoma (M) and näive T cells (M). |
| PI3K -> PIP3 -> Akt AND IL-1β (PI3KAkt_H & IL1) | -> | NF-κB (NfkB_H) | IL-1R signaling activates the NF-κB pathway in a PI3K-dependent mechanism. This co-activation by the interleukin receptor-associated kinase and the recruitment of PI3K has been reported in endothelial and T lymphocytes. | (Bektas et al., 2014; Reddy et al., 1997)⁠. | T limphocytes (H) and epithelial cells (H). |
| ROS (ROS_H) | -> | NF-κB (NfkB_H) | ROS accumulation increase NF-κB pathway activation as a result of two possible mechanisms: the promotion of disulfide-bonds formation favoring NEMO dimerization and S-gluthationylation of IKK-β. | (Finn and Kemp, 2012)⁠ | Pre-B ALL cells (H) |
| TLR1,2,4-10 (TLR_H) | -> | NF-κB (NfkB_H) | TLR1,2,4-10 recognition of their ligands, drive to the recruitment of Myd88 adapter protein whose signaling transduction derives on NF-κB pathway activation. In the most primitive fraction of hematopoietic cells has also been corroborated the expression of functional TLR and their signaling transduction through Myd88. | (Nagai et al., 2006)⁠ | HSPC (M) |
| CXCR4 AND CXCR7 (CXCR4_H & CXCR7_H) | -> | PI3K -> PIP3 -> Akt (PI3KAkt_H) | Both CXCL12 receptors, CXCR4 and CXCR7, are capable of activating PI3K pathway as a result of G-protein and β-arrestin signaling, respectively. The blocking of any of both receptors is sufficient to inhibit Akt phosphorylation, however, the co-stimulation doesn't showed and additive effect. | (Torossian et al., 2014)⁠ | HSPC (H) |
| FoxO3a (FoxO3a_H) | -| | PI3K -> PIP3 -> Akt (PI3KAkt_H) | FoxO3a is involved with PI3K/Akt and ERK, in a negative feedback loop that probed being important for hematopoietic homeostasis. When FoxO3a is inhibited, HSPC show a hyperphosphorylation of Akt. | (Miyamoto et al., 2008)⁠ | HSPC (M) |
| G-CSF (GCSF) | -> | PI3K -> PIP3 -> Akt (PI3KAkt_H) | HPC stimulation with G-CSF increases Akt phosphorylation in serine 473, required for its activation. | (Liu et al., 2007; Vagima et al., 2009)⁠ | HPC (M) and HSPC (H). |
| ROS (ROS_H) | -> | PI3K -> PIP3 -> Akt (PI3KAkt_H) | Under oxidative stress induction, the principal repressor of PI3K pathway called PTEN, is oxidatively inactivated. | (Leslie et al., 2003; Silva et al., 2008)⁠ | Fibroblasts (M), glioblastoma-derived cells (H), macrophages (M) and T-ALL (H). |
| TLR4 (TLR_H) | -> | PI3K -> PIP3 -> Akt (PI3KAkt_H) | On different cellular models, including hematopoietic cells, it has been observed the induction of PI3K pathway after TLR4 stimulation with LPS. TLR stimulation results in the increase of phosphorylated-Akt at threonin 308 and serine 473 by a p38α- and MK2/3-dependent mechanism. | (Guha and Mackman, 2002; McGuire et al., 2013)⁠ | Monocytes (H) and macrophages (M). |
| VLA-4 (VLA4_H) | -> | PI3K -> PIP3 -> Akt (PI3KAkt_H) | Linkage of β1 integrins with some of their ligands, exerts a positive regulation on PI3K activation through the direct interaction with the focal adhesion kinase (FAK) or Pyk2, depending on cell type. | (Melikova et al., 2004)⁠ | HSPC (H) and AML cells (H). |
| FoxO3a (FoxO3a_H) | -| | ROS (ROS_H) | FoxO3a induces the expression of enzymes involved in the regulation of ROS levels, such as superoxide dismutase, catalase and glutathion peroxidases. | (Yalcin et al., 2008)⁠ | HSC (M) |
| IL-1β (IL1) | -> | ROS (ROS_H) | Cellular treatment with IL-1β up-regulates intracellular ROS levels. IL-1β–dependent mechanism for ROS increase is cell-type dependent. For hematopoietic cells, specifically neutrophils, it has been reported that IL-1β promotes NADPH oxidase, while in lymphocytes is through the induction of 5-lipoxygenase. | (Bonizzi et al., 1999; Yagisawa et al., 1995)⁠ | Lymphoid cell lines (H) and neutrophils (H). |
| TLR4 (TLR_H) | -> | ROS (ROS_H) | LPS cellular stimulation upregulates ROS leveles, probably through the induction of NADPH oxidase. | (Hsu and Wen, 2002; Yang et al., 2014)⁠ | Macrophages (M) and whole blood samples (M). |
| CXCR4 (CXCR4_H) | -> | VLA-4 (VLA4_H) | CXCL12 binding to its promoter CXCR4, enhance the high-affinity conformation of VLA-4 to its ligands. | (Hidalgo et al., 2001; Peled et al., 2000; Sanz-Rodríguez et al., 2001) | Myeloma cell line (H), megakaryocytic leukemia cells (H) and HSPC (H). |
| VCAM-1 (VCAM1_H) | -> | VLA-4 (VLA4_H) | VLA-4 integrin binds to VCAM-1 and fibronectin. | (Hidalgo et al., 2001; Peled et al., 2000; Sanz-Rodríguez et al., 2001)⁠ | Myeloma cell line (H), megakaryocytic leukemia cells (H) and HSPC (H). |
