# Supplementary material for: Modeling the Pro-inflammatory Tumor Microenvironment in Acute Lymphoblastic Leukemia Predicts a Breakdown of Hematopoietic-Mesenchymal Communication Networks
Source: Front Physiol. 2016 Aug 19;7:349. doi: 10.3389/fphys.2016.00349 (PMC4990565; doi:10.3389/fphys.2016.00349)
Supplement: Supplementary file 2 [file Table2.doc]

**SUPPLEMENTARY INFORMATION**

Table S2. Public experimental information used for the establishment of the logical rules for the MSC sub-system and soluble microenvironmental factors in the HSPC-MSC model.

| **Input node** | **Type of interaction** | **Target node** | **Bibliographic report** | **Reference** | **Experimental model (H, human; M, mice; R, rat)** |
| --- | --- | --- | --- | --- | --- |
| FoxO3a (FoxO3a_M) | -| | β-catenin (Bcatenin_M) | The accumulation of FOXO transcription factors, compete for β-catenin scaffold activity with T-cell factor, responsible for the transcription of canonical Wnt-pathway targets. | (Crisostomo and Wang, 2008)⁠ | Osteoblasts and osteoblast precursor cells (M). |
| GSK3β (GSK3B_M) | -| | β-catenin (Bcatenin_M) | GSK3β-mediated phosphorylation of β-catenin, triggers its ubiquitination and subsequent proteasome-dependent proteolysis. | (Almeida et al., 2007; Iyer et al., 2013, 2014)⁠ | MSC (M, H). |
| NF-κB (NfkB_M) | -| | β-catenin (Bcatenin_M) | IKKβ, recruited to membrane lipid rafts during NFκB pathway activation, directly phosphorylate the p65 transactivation domain on serine 536 promoting its nuclear translocation. p65 induces the expression of Smurf1 and Smurf2 ('Smad ubiquitin regulatorand factor'), enzymes that increase β-catenin degradation. | (Biver et al., 2014; Malhotra and Kincade, 2009; Zhou, 2011; Zhu et al., 2014)⁠ | MSC (H, M, R) |
| Cx43 (Cx43_M) | -> | CXCL12 (CXCL12_M) | CXCL12 secretion by MSC is dependent on calcium conduction via Cx43 gap junctions mediating intercelular contact. Additionally, transcriptional factor Sp1 nuclear localization is increased, promoting the transcription of *Cxcl12*. | (Cancelas et al., 2000; Schajnovitz et al., 2011)⁠ | Stromal cell line (M), MSC (H) and HSPC (M). |
| β-catenin (Bcatenin_M) | -| | CXCL12 (CXCL12_M) | β-catenin binds to the promoter region of *Cxcl12*, inhibiting its transcription. | (Tamura et al., 2011)⁠ | Stromal cell line (M) |
| G-CSF (GCSF) | -| | CXCL12 (CXCL12_M) | G-CSF down-regulates mRNA levels of CXCL12 and promotes its degradation through the increase of metalloproteinase-9. | (Christopher et al., 2009; Day et al., 2015; Lévesque et al., 2003; Semerad et al., 2005) | Osteoblasts (M) and MSC (M). |
| NF-κB (NfkB_M) | -| | CXCL12 (CXCL12_M) | The activation of canonical NF-κB pathway down-regulates the expression of *Cxcl12* through a RelB-dependent and unknown mechanism. An alternative mechanism considers the participation of miR-146p-5a forming a negative feedback loop between CXCL12 and the NF-κB pathway. | (Hsieh et al., 2013; Madge and May, 2010) | MSC (H), Warthon jelly-MSC (H) and vascular endothelial cell line (H). |
| G-CSF (GCSF) | -> | ERK (ERK_M) | G-CSF treatment, promote an increase in phosphorylation levels of Akt and ERK1/2. | (Furmento et al., 2014)⁠ | Trophoblast cell line (H) |
| ROS (ROS_M) | -> | ERK (ERK_M) | Osteoblasts in vitro treatment with H_2_O_2_ in order to induce oxidative stress, promotes the phosphorylation and activation of ERK1/2. | (Chang et al., 2013)⁠ | Osteoblasts and MSC (rabbit). |
| TLR4 (TLR_M) | -> | ERK (ERK_M) | MSC in vitro stimulation with LPS increases in 40% the concentration of phosphorylated ERK. | (Bai et al., 2004)⁠ | MSC (H) |
| β-catenin (Bcatenin_M) | -> | FoxO3a (FoxO3a_M) | β-catenin interacts directly with FoxO1, FoxO3a and FoxO4 factors, increasing its transcriptional activity. | ⁠(Almeida et al., 2007; Essers et al., 2005; Hoogeboom et al., 2008; Iyer et al., 2014)⁠ | Colorectal adenocarcinoma cell line (H), embrionic kidney cells (M), B lymphoma cels (H), C. elegans and osteoblast precursors (R). |
| ERK (ERK_M) | -| | FoxO3a (FoxO3a_M) | ERK phosphorylates serine 294, 344 and 425 of FoxO3a causing its inactivation and promoting its degradation via MDM2. | (Meng et al., 2010; Yang et al., 2008)⁠⁠ | Lung cancer cell line (H), hepatoma-derived cells (H), and embrionic fibroblasts (M), |
| PI3K -> PIP3 -> Akt (PI3KAkt_M) | -| | FoxO3a (FoxO3a_M) | Akt phosphorylate threonine 32, serine 253 and serine 315 of FoxO3a promoting its cytoplasmic localization and down-regulating its target genes. | (Brunet et al., 1999; Meng et al., 2010; Shankar et al., 2013) | Kidney epithelial cells (H), lung fibroblasts (chinese hamster), lung cancer and pancreatic cancer cells (H). |
| ROS (ROS_M) | -> | FoxO3a (FoxO3a_M) | Accumulation of ROS promote the nuclear localization of FOXO, including FoxO3a, increasing the transcription of ROS-regulating enzymes, such as catalase. | (Almeida et al., 2007; Essers et al., 2005; Gorbunov et al., 2013)⁠ | Osteblast precursors (M), C. elegans and MSC (M) |
| IL-1α and IL1β (IL1) | -> | G-CSF (GCSF) | IL-1α treatment showed an increased expression of G-CSF. Considering that both IL-1α and IL-1β bind and activate the same receptor triggering Myd88 signaling, G-CSF could be induced in MSC or other stromal components expressing IL-1RI, like endothelial cells. | (Allakhverdi et al., 2013; Boettcher et al., 2014; Majumdar et al., 2000)⁠ | MSC (H) |
| PI3K -> PIP3 -> Akt (PI3KAkt_M) | -| | GSK3β (GSK3B_M) | Akt activation phosphorylates and inactivate GSK3β. | (Biver et al., 2014; Case et al., 2011)⁠ | MSC (H, M). |
| NF-κB (NfkB_M) | -> | IL-1β (IL1) | In silico analysis showed that IL-1β promoter region contains binding sites for NF-κB pathway elements. | (Higashikuni et al., 2013; Wang et al., 2002)⁠ | Cardiomyocytes (R), fibroblasts (R), endothelial cells (H), in silico (H,M), synovial fibroblasts (M). |
| PI3K -> PIP3 -> Akt (PI3KAkt_M) | -| | IL-1β (IL1) | Inhibition of PI3K pathway elements results in an increase on IL-1β plasma levels in mice with polymicrobial sepsis. This could be explained through the PI3K/Akt-mediated increase on the production of antagonist molecules for the receptor for IL-1. | (Li and Smith, 2014; Williams et al., 2004)⁠ | Fibrocytes (H), intra-abdominal sepsis model (M) |
| ROS (ROS_M) | -> | IL-1β (IL1) | The induction of ROS accumulation in MSC, increase IL-1β expression and secretion. | (Yang et al., 2010)⁠ | MSC (R) |
| ROS AND ERK (ROS_M & ERK_M) | -> | NF-κB (NfkB_M) | H_2_O_2_-dependent activation of the NF-κB pathway, requires the co-paticipation of ERK for IκBα phosphorylation and degradation. | (Bai et al., 2004)⁠ | MSC (rabbit) |
| PI3K -> PIP3 -> Akt AND (IL-1α OR IL-1β) (PI3KAkt_M & IL1) | -> | NF-κB (NfkB_M) | MyD88-dependent IL-1R signaling activates NF-κB pathway. In endothelial cells, it has been reported that this NF-κB activation requires PI3K, recruited through the interaction of p85 with IL-1R. | (Carrero et al., 2012; Reddy et al., 1997; Sizemore et al., 1999)⁠ | MSC (H) and epithelial cell line (H). |
| TLR4 (TLR_M) | -> | NF-κB (NfkB_M) | TLR4 recognition of its ligand, drives to the recruitment of Myd88 adapter protein whose signaling transduction derives on NF-κB pathway activation. | (Crisostomo and Wang, 2008; Wang et al., 2009)⁠ | MSC (H) |
| FoxO3a (FoxO3a_M) | -| | ROS (ROS_M) | FoxO3a induces the expression of enzymes involved in the regulation of ROS levels, such as superoxide dismutase and catalase. | (Almeida et al., 2007; Liu et al., 2005)⁠⁠ | Epithelial cell line (H) and osteoblast precursors (M). |
| IL-1β (IL1) | -> | ROS (ROS_M) | MSC treatment with IL-1β up-regulates intracellular ROS levels. | (Liu et al., 2013)⁠ | MSC (M) |
| TLR4 (TLR_M) | -> | ROS (ROS_M) | Cellular stimulation with LPS increase intracellular ROS levels. In other models it has been observed that this effect is dependent on the increase of NADPH oxidase concentration. | (Gorbunov et al., 2013)⁠ | MSC (M) |
| G-CSF (GCSF) | -> | PI3K -> PIP3 -> Akt (PI3KAkt_M) | G-CSF binding to its receptor in trophoblast cells, increases the levels of phosphorylated-Akt and ERK, co-participating in the promotion of MMP-2 expression. The G-CSF- dependent promotion of MMP-2, has been also reported on MSC, so we inferred that the Akt and ERK activation are also downstream G-CSF receptor. | (Furmento et al., 2014; Ponte et al., 2012)⁠ | Trophoblast cell line (H) and MSC (H). |
| ROS (ROS_M) | -> | PI3K -> PIP3 -> Akt (PI3KAkt_M) | Oxidative stress inhibits the principal repressor of PI3K pathway, PTEN. | (Buldak et al., 2013; Lee et al., 2002)⁠ | Epithelial cell line (H) |
| TLR4 (TLR_M) | -> | PI3K -> PIP3 -> Akt (PI3KAkt_M) | Phosphorylation of Akt in serine 473 after LPS stimulation, exhert a protective effect enhancing cellular survival. | (Wang et al., 2009)⁠ | MSC (M) |
| β-catenin (Bcatenin_M) | -| | VCAM-1 (VCAM1_M) | MSC treatment with the canonical Wnt pathway ligand, Wnt3a, affects cellular adhesion and morphology through the down-regulation of VCAM-1 expression. | (Malhotra and Kincade, 2009)⁠ | MSC (M) |
| NF-κB (NfkB_M) | -> | VCAM-1 (VCAM1_M) | NF-κB participates in the up-regulation of VCAM-1 expression, probably directly binding to NF-κB sites in the promoter region of the VCAM-1 gene. | (Bayat et al., 2007; Hu et al., 2013; Malhotra and Kincade, 2009)⁠ | Vascular endothelial cell line (H) and MSC (M, R) |
| PI3K -> PIP3 -> Akt (PI3KAkt_M) | -> | VCAM-1 (VCAM1_M) | Cellular treatment with a specific PI3K inhibitor, showed that this pathway is involved in the up-regulation of VCAM-1 expression. | (Hu et al., 2013)⁠ | MSC (R) |
