# Supplementary material for: Modeling the Pro-inflammatory Tumor Microenvironment in Acute Lymphoblastic Leukemia Predicts a Breakdown of Hematopoietic-Mesenchymal Communication Networks
Source: Front Physiol. 2016 Aug 19;7:349. doi: 10.3389/fphys.2016.00349 (PMC4990565; doi:10.3389/fphys.2016.00349)
Supplement: Supplementary file 3 [file Table3.doc]

**SUPPLEMENTARY INFORMATION**

Table S3. Common names and genes ID in *Homo sapiens* for represented molecules in the network nodes. NA, no gene ID apply.

| **Node** | **Common name** | **Gene ID (*Homo sapiens*)** |
| --- | --- | --- |
| Cx43_M | Connexin 43 | GJA1 |
| lTLR | Lipopolysaccharide, O-antigen | NA, synthesized by Gram-negative bacterias |
| Bcatenin_H, Bcatenin_M | β-catenin | CTNNB1 |
| CXCL12_M | Stromal-cell derived factor 1, C-X-C motif chemokine 12, CXCL12, SDF-1 | CXCL12 |
| CXCR4_H | C-X-C chemokine receptor type 4, CXCR4 | CXCR4 |
| CXCR7_H | C-X-C chemokine receptor type 7, CXCR7 | ACKR3 |
| ERK_H, ERK_M | Extracellular signal-regulated kinase, ERK | MAPK1 |
| FoxO3a_H, FoxO3a_M | Forkhead box O3, FoxO3a | FOXO3 |
| GCSF | Granulocyte-colony stimulating factor, G-CSF | CSF3 |
| Gfi1_H | Growth factor independent 1, Gfi1 | GFI1 |
| GSK3B_H, GSK3B_M | Glycogen synthase kinase 3β, GSK3β | GSK3B |
| IL1 | Interleukin-1, IL-1α, IL-1β | IL1A, IL1B |
| NfkB_H, NfkB_M | Necrosis factor-κB, NF-κB | NFKB1 |
| ROS_H, ROS_M | Reactive oxygen species | NA, product of cellular metabolism |
| PI3KAkt_H, PI3KAkt_M | Phosphoinositide 3-kinase, PI3K  Protein kinase B, PKB, Akt | PIK3CB  AKT1 |
| TLR_H, TLR_M | Toll-like receptor 4 | TLR4 |
| VCAM1_M | Vascular cell adhesion molecule-1, VCAM-1 | VCAM1 |
| VLA4_H | Very late antigen-4, VLA-4 (integrin dimer composed by alpha 4 and beta 1 subunits) | ITGA4  ITGB1 |
