# Supplementary material for: Modeling the Pro-inflammatory Tumor Microenvironment in Acute Lymphoblastic Leukemia Predicts a Breakdown of Hematopoietic-Mesenchymal Communication Networks
Source: Front Physiol. 2016 Aug 19;7:349. doi: 10.3389/fphys.2016.00349 (PMC4990565; doi:10.3389/fphys.2016.00349)
Supplement: Supplementary file 4 [file Table4.docx]

**SUPPLEMENTARY INFORMATION**

Table S4. Referenced experimental observations used for the mutant simulations analysis.

| **Reference** | **Experimental observation** | **Associated model node** |
| --- | --- | --- |
| Cobas et al., 2004. | Induced inactivation of β-catenin gene in mice hematopoietic precursors, does not impair bone marrow reconstitution for all major blood lineages. | Bcatenin_H |
| Jeannet et al., 2008. | β- and γ-catenin ablated mice hematopoietic cells, sustain their capacity for a normal long-term multilineage hematopoietic reconstitution. | Bcatenin_H |
| Greenbaum et al., 2013. | The inhibition of CXCL12 expression by osteoblasts and MSCs in mice model, induce reduction in bone marrow cellularity, but increase of HPCs in blood and spleen. | CXCL12_M |
| Tzeng et al., 2011. | CXCL12 knockout mice leads to a reduction in bone marrow HSC numbers and B cell progenitors. However, they show an increase in other hematopoietic progenitor cells in BM, spleen and mostly in peripheral blood. | CXCL12_M |
| Sugiyama et al., 2006. | CXCR4 conditionally deficient mice, showed a decrease in HSC population. Sugiyama and collaborators suggest that the observations may be due to microenvironmental modifications that enhance the expansion of progenitor hematopoietic cells. | CXCR4_H |
| Sierro et al., 2007. | B cells and granulocytes development in fetal liver, appears to be unaffected on CXCR7 knockout mouse. | CXCR7_H |
| Hock et al., 2004. | HSCs from Gfi1-/- mice show increased proliferation rates and are out-competed in competitive repopulation assays with wild-type cells. | Gfi1_H |
| Zeng et al., 2004. | HSCs from Gfi1 knockout mice, show increased proliferation rates producing a severe reduction in HSC and CLP population. Interestingly, bone marrow transplantation from Gfi1 defficient mice, have similar reconstitution capacities at day 8, but impaired until day 12. | Gfi1_H |
| Holmes et al., 2008. | GSK3β inhibition, reduce HSPC population expansion enriched from umbilical cord blood. | GSK3B_H |
| Champelovier et al., 2008. | Inhibition of PI3K in human leukemia-derived cell lines, resulted in an antiproliferative and pro-apoptotic effect. | PI3KAkt_H |
| Williams et al., 2004. | PI3K plays a regulatory role on pro-inflammatory cytokine production analyzed in an experimental mice model of polymicrobial sepsis. | PI3KAkt_H, PI3KAkt_M |
| Xu et al., 2012. | PI3K is involved in promoting MSC survival and reduction of ROS production under oxidative stress. | PI3KAkt_M |
| Scott et al., 2003. | VLA-4 conditional inhibition in mice hematopoietic progenitor cells increase their levels in blood and spleen, and also showed and impair bone marrow homing capacity. | VLA-4, VCAM1_M |
| Wang et al., 1998. | VCAM-1 interaction with VLA-4 expressed by HSPC, plays a role in ex vivo apoptosis inhibition and proliferation enhancement of the primitive population of hematopoietic cells. | VLA-4, VCAM1_M |
| Satija et al., 2013. | Lithium mediated inhibition of GSK3β in human MSC, induce a down-regulation in CXCL12 transcription. | GSK3B_M |

| Kode et al., 2014. | Mice with constitutive activation of β-catenin in osteoblasts show a decrease in HSPC population and develop AML with leukemia initiatin cells in among an increased LT-HSC population. | Bcatenin_M |
| --- | --- | --- |
| Cortez et al., 2013. | Rats fed with a high-fat diet, increase an NF-κB-dependent IL- 1 increased production. They also observed hyperplasia with an increase in the numbers of granulocytic cells. | NfkB_M |
| Kirstetter et al., 2006. | The conditional expression of β-catenin in adult mice hematopoietic cells induce an initial increase in bone marrow celularity followed by hematopoietic failure, which was assocatied to the lost of HSC repopulation capacity and a block in myeloid and lymphoid development. | Bcatenin_H |
| Yamazaki et al., 2006. | FoxO3a activation and nuclear accumulation induces HSCs hibernation or progenitor cells apoptosis. | FoxO3a_H |
| Khandanpour et al., 2013. | Gfi1 is an accelerator of T-ALL development in different mice model, but its overexpression alone is also capable of inducing the leukemic phenotype. The ablation of this zinc finger transcription factor, increases the apoptosis of T-ALL cells. | Gfi1_H |
| Wang et al., 2013. | The co-inhibition of PI3K and β-catenin, both up-regulated in AML cell line, decrease tumor growth in a xenotransplant mice model. | PI3KAkt_H |
| Lu et al., 2013. | Free iron produces increased levels of ROS responsible for a decrease in blood cells production and, MSC apoptosis induction and reduced proliferation. | ROS_H, ROS_M |
| Zhang et al., 2015. | Iron oxidative damage is associated to a PI3K-dependent decrease of MSCs FoxO3a activation and a diminish in chemokines production, including CXCL12. | ROS_M |
| Shalapour et al., 2011. | High VLA-4 expression in a subset of B-ALL patients, is associated with disregulation of PI3K/Akt and Wnt pathways associated genes. | VLA-4 |
| Schofield et al., 1998. | Increased VLA-4 binding to the extracellular matrix component, fibronectin, promotes HSPC population expansion. | VLA-4 |
