# Supplementary material for: Modeling the Pro-inflammatory Tumor Microenvironment in Acute Lymphoblastic Leukemia Predicts a Breakdown of Hematopoietic-Mesenchymal Communication Networks
Source: Front Physiol. 2016 Aug 19;7:349. doi: 10.3389/fphys.2016.00349 (PMC4990565; doi:10.3389/fphys.2016.00349)
Supplement: Supplementary file 5 [file Presentation1.pdf]

REFERENCES

Allakhverdi, Z., Comeau, M. R., Armant, M., Agrawal, R., Woodfolk, J. A, Sehmi R., et al. (2013). Mast cell-activated bone marrow mesenchymal stromal cells regulate proliferation and lineage commitment of CD34+ progenitor cells. *Front. Immunol.* 4. doi:10.3389/fimmu.2013.00461.

Almeida, M., Han, L., Martin-Millan, M., O'Brien, C. A., and Manolagas, S. C. (2007). Oxidative stress antagonizes Wnt signaling in osteoblast precursors by diverting beta-catenin from T cell factor- to forkhead box O-mediated transcription. *J. Biol. Chem.* 282, 27298–305. doi:10.1074/jbc.M702811200.

Bai, X., Lu, D., Bai, J., Zheng, H., Ke, Z., Li, X., et al. (2004). Oxidative stress inhibits osteoblastic differentiation of bone cells by ERK and NF-κB. *Biochem. Biophys. Res. Commun.* 314, 197–207. doi:10.1016/j.bbrc.2003.12.073.

Bayat, H., Xu, S., Pimentel, D., Cohen, R. A., and Jiang, B. (2007). Activation of Thromboxane Receptor Upregulates Interleukin (IL)-1β−Induced VCAM-1 Expression Through JNK Signaling. *Arterioscler. Thromb. Vasc. Biol.* 28, 127–134. doi:10.1161/ATVBAHA.107.150250.

Bektas, A., Zhang, Y., Lehmann, E., Iii, W. H. W., Becker, K. G., Ferrucci, L., et al. (2014). Age- associated changes in basal NF- κB function in human CD4+ T lymphocytes via dysregulation of PI3 kinase. *Aging (Albany. NY).* 6, 957–974.

Biver, E., Thouverey, C., Magne, D., and Caverzasio, J. (2014). Crosstalk between tyrosine kinase receptors, GSK3 and BMP2 signaling during osteoblastic differentiation of human mesenchymal stem cells. *Mol. Cell. Endocrinol.* 382, 120–130. doi:10.1016/j.mce.2013.09.018.

Boettcher, S., Gerosa, R. C., Radpour, R., Bauer, J., Ampenberger, F., Heikenwalder, M., et al. (2014). Endothelial cells translate pathogen signals into G-CSF-driven emergency granulopoiesis. *Blood* 124, 1393–1403. doi:10.1182/blood-2014-04-570762.

Bonizzi, G., Piette, J., Schoonbroodt, S., Greimers, R., Havard, L., Merville, M. P., et al. (1999). Reactive oxygen intermediate-dependent NF-kappaB activation by interleukin-1beta requires 5-lipoxygenase or NADPH oxidase activity. *Mol. Cell. Biol.* 19, 1950–1960.

Brunet, A., Bonni, A., Zigmond, M. J., Lin, M. Z., Juo, P., Hu, L. S., et al. (1999). Akt Promotes Cell Survival by Phosphorylating and Inhibiting a Forkhead Transcription Factor. *Cell* 96, 857–868.

Brunet, A., Sweeney, L. B., Sturgill, J. F., Katrin, F., Greer, P. L., Lin, Y., et al. (2004). Stress-Dependent Regulation of FOXO Transcription Factors by the SIRT1 Deacetylase. *Science (80-.).* 303, 2011–2016.

Buldak, R. J., Polaniak, R., Buldak, L., Mielanczyk, L., Kukla, M., Skonieczna, M., et al. (2013). Exogenous administration of visfatin affects cytokine secretion and increases oxidative stress in human malignant melanoma ME45 cells. *J. Physiol. Pharmacol.* 64, 377–385.

Burns, J. M., Summers, B. C., Wang, Y., Melikian, A., Berahovich, R., Miao, Z., et al. (2006). A novel

chemokine receptor for SDF-1 and I-TAC involved in cell survival, cell adhesion, and tumor development. *J. Exp. Med.* 203, 2201–13. doi:10.1084/jem.20052144.

Cancelas, J. a, Koevoet, W. L., de Koning, a E., Mayen, a E., Rombouts, E. J., and Ploemacher, R. E. (2000). Connexin-43 gap junctions are involved in multiconnexin-expressing stromal support of hemopoietic progenitors and stem cells. *Blood* 96, 498–505. Available at: http://www.ncbi.nlm.nih.gov/pubmed/10887111.

Carrero, R., Cerrada, I., Lledó, E., Dopazo, J., García-García, F., Rubio, M.-P., et al. (2012). IL1β induces mesenchymal stem cells migration and leucocyte chemotaxis through NF-κB. *Stem Cell Rev.* 8, 905–16. doi:10.1007/s12015-012-9364-9.

Case, N., Thomas, J., Sen, B., Styner, M., Xie, Z., Galior, K., et al. (2011). Mechanical regulation of glycogen synthase kinase 3β (GSK3β) in mesenchymal stem cells is dependent on Akt protein serine 473 phosphorylation via mTORC2 protein. *J. Biol. Chem.* 286, 39450–39456. doi:10.1074/jbc.M111.265330.

Champelovier, P., El Atifi, M., Pautre, V., Rostaing, B., Berger, F., and Seigneurin, D. (2008). Specific inhibition of basal mitogen-activated protein kinases and phosphatidylinositol 3 kinase activities in leukemia cells: a possible therapeutic role for the kinase inhibitors. *Exp. Hematol.* 36, 28–36. doi:10.1016/j.exphem.2007.08.027.

Chang, J., Liu, F., Lee, M., Wu, B., Ting, K., Zara, J. N., et al. (2013). NF- κ B inhibits osteogenic differentiation of mesenchymal stem cells by promoting β -catenin degradation. *Proc. Natl. Acad. Sci. U. S. A.* 110, 9469–9474. doi:10.1073/pnas.1300532110.

Christopher, M. J., Liu, F., Hilton, M. J., Long, F., and Link, D. C. (2009). Suppression of CXCL12 production by bone marrow osteoblasts is a common and critical pathway for cytokine-induced mobilization. *Blood* 114, 1331–1339. doi:10.1182/blood-2008-10-184754.

Cobas, M., Wilson, A., Ernst, B., Mancini, S. J. C., MacDonald, H. R., Kemler, R., et al. (2004). Beta-catenin is dispensable for hematopoiesis and lymphopoiesis. *J. Exp. Med.* 199, 221–229. doi:10.1084/jem.20031615.

Coggins, N. L., Trakimas, D., Chang, S. L., Ehrlich, A., Ray, P., Luker, K. E., et al. (2014). CXCR7 controls competition for recruitment of β-arrestin 2 in cells expressing both CXCR4 and CXCR7. *PLoS One* 9, e98328. doi:10.1371/journal.pone.0098328.

Cortez, M., Carmo, L. S., Rogero, M. M., Borelli, P., and Fock, R. A. (2013). A high-fat diet increases IL-1, IL-6, and TNF-α production by increasing NF-κb and attenuating PPAR-γ expression in bone marrow mesenchymal stem cells. *Inflammation* 36, 379–386. doi:10.1007/s10753-012-9557-z.

Crisostomo, P., and Wang, Y. (2008). Human mesenchymal stem cells stimulated by TNFa, LPS, or hypoxia produce growth factors by an NFkB-but not JNK-dependent mechanism. *Am. J. ...* 46202, 675–682. doi:10.1152/ajpcell.00437.2007.

Day, R. B., Bhattacharya, D., Nagasawa, T., and Link, D. C. (2015). Granulocyte colony-stimulating factor reprograms bone marrow stromal cells to actively suppress B lymphopoiesis in mice. *Blood*, pii: blood–2015–02–629444. Available at: http://www.bloodjournal.org/content/early/2015/03/26/blood-2015-02-629444.abstract.

De La Luz Sierra, M., Gasperini, P., McCormick, P. J., Zhu, J., and Tosato, G. (2007). Transcription factor Gfi-1 induced by G-CSF is a negative regulator of CXCR4 in myeloid cells. *Blood* 110, 2276–85. doi:10.1182/blood-2007-03-081448.

De la Luz Sierra, M., Sakakibara, S., Gasperini, P., Salvucci, O., Jiang, K., McCormick, P. J., et al. (2010). The transcription factor Gfi1 regulates G-CSF signaling and neutrophil development through the Ras activator RasGRP1. *Blood* 115, 3970–9. doi:10.1182/blood-2009-10-246967.

Delgado-Martín, C., Escribano, C., Pablos, J. L., Riol-Blanco, L., and Rodríguez-Fernández, J. L. (2011). Chemokine CXCL12 uses CXCR4 and a signaling core formed by bifunctional akt, Extracellular Signal-regulated Kinase (ERK)1/2, and Mammalian Target of Rapamycin Complex 1 (mTORC1) proteins to control chemotaxis and survival simultaneously in mature dendriti. *J. Biol. Chem.* 286, 37222–37236. doi:10.1074/jbc.M111.294116.

Doan, L. L., Porter, S. D., Duan, Z., Flubacher, M. M., Montoya, D., Tsichlis, P. N., et al. (2004). Targeted transcriptional repression of Gfi1 by GFI1 and GFI1B in lymphoid cells. *Nucleic Acids Res.* 32, 2508–2519. doi:10.1093/nar/gkh570.

Essers, M. a G., de Vries-Smits, L. M. M., Barker, N., Polderman, P. E., Burgering, B. M. T., and Korswagen, H. C. (2005). Functional interaction between beta-catenin and FOXO in oxidative stress signaling. *Science* 308, 1181–4. doi:10.1126/science.1109083.

Finn, N. A., and Kemp, M. L. (2012). Pro-oxidant and antioxidant effects of N-acetylcysteine regulate doxorubicin-induced NF-kappa B activity in leukemic cells. *Mol. Biosyst.* 8, 650–62. doi:10.1039/c1mb05315a.

Furmento, V. a., Marino, J., Blank, V. C., and Roguin, L. P. (2014). The granulocyte colony-stimulating factor (G-CSF) upregulates metalloproteinase-2 and VEGF through PI3K/Akt and Erk1/2 activation in human trophoblast Swan 71 cells. *Placenta* 35, 937–946. doi:10.1016/j.placenta.2014.09.003.

Gabelloni, M. L., Sabbione, F., Jancic, C., Bass, J. F., Keitelman, I., Iula, L., et al. (2013). NADPH oxidase derived reactive oxygen species are involved in human neutrophil IL-1β secretion but not in inflammasome activation. *Eur. J. Immunol.* 43, 3324–3335. doi:10.1002/eji.201243089.

Gorbunov, N. V., Garrison, B. R., McDaniel, D. P., Zhai, M., Liao, P. J., Nurmemet, D., et al. (2013). Adaptive redox response of mesenchymal stromal cells to stimulation with lipopolysaccharide inflammagen: Mechanisms of remodeling of tissue barriers in sepsis. *Oxid. Med. Cell. Longev.* 2013. doi:10.1155/2013/186795.

Greenbaum, A., Hsu, Y.-M. S., Day, R. B., Schuettpelz, L. G., Christopher, M. J., Borgerding, J. N., et

al. (2013). CXCL12 in early mesenchymal progenitors is required for haematopoietic stem-cell maintenance. *Nature* 495, 227–30. doi:10.1038/nature11926.

Griffith, C. E., Zhang, W., and Wange, R. L. (1998). ZAP-70-dependent and -independent Activation of Erk in. *J. Biol. Chem.* 273, 10771–10776.

Guha, M., and Mackman, N. (2002). The phosphatidylinositol 3-kinase-Akt pathway limits lipopolysaccharide activation of signaling pathways and expression of inflammatory mediators in human monocytic cells. *J. Biol. Chem.* 277, 32124–32132. doi:10.1074/jbc.M203298200.

Hidalgo, A., Sanz-Rodríguez, F., Rodríguez-Fernández, J. L., Albella, B., Blaya, C., Wright, N., et al. (2001). Chemokine stromal cell-derived factor-1α modulates VLA-4 integrin-dependent adhesion to fibronectin and VCAM-1 on bone marrow hematopoietic progenitor cells. *Exp. Hematol.* 29, 345–355. doi:10.1016/S0301-472X(00)00668-8.

Higashikuni, Y., Tanaka, K., Kato, M., Nureki, O., Hirata, Y., Nagai, R., et al. (2013). Toll-like receptor-2 mediates adaptive cardiac hypertrophy in response to pressure overload through interleukin-1β upregulation via nuclear factor κB activation. *J. Am. Heart Assoc.* 2, e000267. doi:10.1161/JAHA.113.000267.

Hock, H., Hamblen, M. J., Rooke, H. M., Schindler, J. W., Saleque, S., Fujiwara, Y., et al. (2004). Gfi-1 restricts proliferation and preserves functional integrity of haematopoietic stem cells. *Nature* 431, 1002–1007. doi:10.1038/nature02994.

Holmes, T., O'Brien, T. a, Knight, R., Lindeman, R., Shen, S., Song, E., et al. (2008). Glycogen synthase kinase-3beta inhibition preserves hematopoietic stem cell activity and inhibits leukemic cell growth. *Stem Cells* 26, 1288–1297. doi:10.1634/stemcells.2007-0600.

Hoogeboom, D., Essers, M. A. G., Polderman, P. E., Voets, E., Smits, L. M., and Burgering, B. Mt. (2008). Interaction of FOXO with β-Catenin Inhibits β-Catenin/T Cell Factor Activity. *J. Biol. Chem.* 283, 9224–9230. doi:10.1074/jbc.M706638200.

Hsieh, J., Huang, T., Cheng, S., Lin, W., Tsai, T., Lee, O. K., et al. (2013). miR-146a-5p circuitry uncouples cell proliferation and migration, but not differentiation, in human mesenchymal stem cells. *Nucleic Acids Res.* 41, 9753–9763. doi:10.1093/nar/gkt666.

Hsu, H.-Y. Y., and Wen, M.-H. H. (2002). Lipopolysaccharide-mediated reactive oxygen species and signal transduction in the regulation of interleukin-1 gene expression. *J. Biol. Chem.* 277, 22131–22139. doi:10.1074/jbc.M111883200.

Hu, Y., Cheng, P., Jiang-chun, M., Xue, Y., and Liu, Y. H. (2013). Platelet-derived growth factor BB mediates the glioma-induced migration of bone marrow-derived mesenchymal stem cells by promoting the expression of vascular cell adhesion molecule-1 through the PI3K, P38 MAPK and NF-kB pathways. *Oncol. Rep.* 30, 2755–2764. doi:10.3892/or.2013.2780.

Iyer, S., Ambrogini, E., Bartell, S. M., Han, L., Roberson, P. K., Cabo, R. De, et al. (2013). FOXOs

attenuate bone formation by suppressing Wnt signaling. *J. Clin. Invest.* 123, 3409–3419. doi:10.1172/JCI68049.al.

Iyer, S., Han, L., Bartell, S. M., Kim, H.-N., Gubrij, I., de Cabo, R., et al. (2014). Sirtuin1 (Sirt1) promotes cortical bone formation by preventing β-catenin sequestration by FoxO transcription factors in osteoblast progenitors. *J. Biol. Chem.* 289, 24069–78. doi:10.1074/jbc.M114.561803.

Jeannet, G., Scheller, M., Scarpellino, L., Duboux, S., Gardiol, N., Back, J., et al. (2008). Long-term, multilineage hematopoiesis occurs in the combined absence of β-catenin and γ-catenin. *Blood* 111, 142–149. doi:10.1182/blood-2007-07-102558.

Jones, B. W., Means, T. K., Heldwein, K. a, Keen, M. a, Hill, P. J., Belisle, J. T., et al. (2001). Different Toll-like receptor agonists induce distinct macrophage responses. *J. Leukoc. Biol.* 69, 1036–1044.

Keshari, R. S., Verma, A., Barthwal, M. K., and Dikshit, M. (2013). Reactive oxygen species-induced activation of ERK and p38 MAPK mediates PMA-induced NETs release from human neutrophils. *J. Cell. Biochem.* 114, 532–40. doi:10.1002/jcb.24391.

Khandanpour, C., Sharif-askari, E., Vassen, L., Gaudreau, M., Zhu, J., Paul, W. E., et al. (2010). Evidence that Growth factor independence 1b regulates dormancy and peripheral blood mobilization of hematopoietic stem cells Evidence that Growth factor independence 1b regulates dormancy and peripheral blood mobilization of hematopoietic stem cells. *Blood* 116, 5149–5161. doi:10.1182/blood-2010-04-280305.

Kirstetter, P., Anderson, K., Porse, B. T., Jacobsen, S. E. W., and Nerlov, C. (2006). Activation of the canonical Wnt pathway leads to loss of hematopoietic stem cell repopulation and multilineage differentiation block. *Nat. Immunol.* 7, 1048–1056. doi:10.1038/ni1381.

Kode, A., Manavalan, J. S., Mosialou, I., Bhagat, G., Rathinam, C. V, Luo, N., et al. (2014). Leukemogenesis Induced by an Activating β-catenin mutation in Osteoblasts. *Nature* 506, 240–244. doi:10.1038/nature11130.Reduced.

Kumar, R., Tripathi, V., Ahmad, M., Nath, N., Mir, R. A., Chauhan, S. S., et al. (2012). CXCR7 mediated Giα independent activation of ERK and Akt promotes cell survival and chemotaxis in T cells. *Cell. Immunol.* 272, 230–241. doi:10.1016/j.cellimm.2011.09.015.

Lee, S. R., Yang, K. S., Kwon, J., Lee, C., Jeong, W., and Rhee, S. G. (2002). Reversible inactivation of the tumor suppressor PTEN by H2O2. *J. Biol. Chem.* 277, 20336–20342. doi:10.1074/jbc.M111899200.

Leslie, N. R., Bennett, D., Lindsay, Y. E., Stewart, H., Gray, A., and Downes, C. P. (2003). Redox regulation of PI 3-kinase signalling via inactivation of PTEN. *EMBO J.* 22, 5501–5510. doi:10.1093/emboj/cdg513.

Lévesque, J., Hendy, J., Takamatsu, Y., Simmons, P. J., and Bendall, L. J. (2003). Disruption of the

CXCR4 / CXCL12 chemotactic interaction during hematopoietic stem cell mobilization induced by GCSF or cyclophosphamide. *J. Clin. Invest.* 111, 187–196. doi:10.1172/JCI200315994.Introduction.

Li, B., and Smith, T. J. (2014). PI3K/AKT pathway mediates induction of IL-1RA by TSH in fibrocytes: Modulation by PTEN. *J. Clin. Endocrinol. Metab.* 99, jc20141257. doi:10.1210/jc.2014-1257.

Lin, L., Hron, J. D., and Peng, S. L. (2004). Regulation of NF- kB , Th activation, and autoinflammation by the Forkhead transcription factor Foxo3a. *Immunity* 21, 203–213.

Liu, H., Mihara, K., and Song, G. (2007). Interferon- γ attenuates the survival activity of G-CSF through PI3K / Akt signaling pathway in mouse multipotent progenitor cells. *Ann. Hematol.* 86, 547–555. doi:10.1007/s00277-007-0308-4.

Liu, J.-W., Chandra, D., Rudd, M. D., Butler, A. P., Pallotta, V., Brown, D., et al. (2005). Induction of prosurvival molecules by apoptotic stimuli: involvement of FOXO3a and ROS. *Oncogene* 24, 2020–2031. doi:10.1038/sj.onc.1208385.

Liu, Q., Jin, L., Shen, F. H., Balian, G., and Li, X. J. (2013). Fullerol nanoparticles suppress inflammatory response and adipogenesis of vertebral bone marrow stromal cells--a potential novel treatment for intervertebral disc degeneration. *Spine J.* 13, 1571–80. doi:10.1016/j.spinee.2013.04.004.

Lu, W., Zhao, M., Rajbhandary, S., Xie, F., Chai, X., Mu, J., et al. (2013). Free iron catalyzes oxidative damage to hematopoietic cells/mesenchymal stem cells in vitro and suppresses hematopoiesis in iron overload patients. *Eur. J. Haematol.* 91, 249–261. doi:10.1111/ejh.12159.

Madge, L. a., and May, M. J. (2010). Classical NF-kB activation negatively regulates noncanonical NF-kB-dependent CXCL12 expression. *J. Biol. Chem.* 285, 38069–38077. doi:10.1074/jbc.M110.147207.

Majumdar, M. K., Thiede, M. A., Haynesworth, S. E., Bruder, S. P., and Gerson, S. L. (2000). Human Marrow-Derived Mesenchymal Stem Cells (MSCs) Express Hematopoietic Cytokines and Support Long-Term Hematopoiesis When Differentiated Toward Stromal and Osteogenic Lineages. *J. Hematother. Stem Cell Res.* 9, 841–848.

Malhotra, S., and Kincade, P. W. (2009). Canonical Wnt pathway signaling suppresses VCAM-1 expression by marrow stromal and hematopoietic cells. *Exp. Hematol.* 37, 19–30. doi:10.1016/j.exphem.2008.08.008.Canonical.

McGilvray, I. D., Lu, Z., Bitar, R., Dackiw, A. P. B., Davreux, C. J., and Rotstein, O. D. (1997). VLA-4 Integrin Cross-linking on Human Monocytic THP-1 Cells Induces Tissue Factor Expression by a Mechanism Involving Mitogen-activated Protein Kinase VLA-4 Integrin Cross-linking on Human Monocytic THP-1 Cells Induces Tissue Factor Expression by a Mechan. *J. Biol. Chem.* 272, 10287–10294. doi:10.1074/jbc.272.15.10287.

McGuire, V. a, Gray, A., Monk, C. E., Santos, S. G., Lee, K., Aubareda, A., et al. (2013). Cross talk between the Akt and p38α pathways in macrophages downstream of Toll-like receptor signaling. *Mol. Cell. Biol.* 33, 4152–65. doi:10.1128/MCB.01691-12.

Melikova, S., Dylla, S. J., and Verfaillie, C. M. (2004). Phosphatidylinositol-3-kinase activation mediates proline-rich tyrosine kinase 2 phosphorylation and recruitment to β1-integrins in human CD34 + cells. *Exp. Hematol.* 32, 1051–1056. doi:10.1016/j.exphem.2004.07.018.

Melo, R., Longhini, A. L., Louzao Bigarella, C., Ozello Baratti, M., Traina, F., Favaro, P., et al. (2014). CXCR7 Is Highly Expressed in Acute Lymphoblastic Leukemia and Potentiates CXCR4 Response to CXCL12. *PLoS One* 9, e85926. doi:10.1371/journal.pone.0085926.

Meng, J., Fang, B., Liao, Y., Chresta, C. M., Smith, P. D., and Roth, J. A. (2010). Apoptosis Induction by MEK Inhibition in Human Lung Cancer Cells Is Mediated by Bim. *PLoS One* 5, e13026. doi:10.1371/journal.pone.0013026.

Miyamoto, K., Miyamoto, T., Kato, R., Yoshimura, A., Motoyama, N., and Suda, T. (2008). FoxO3a regulates hematopoietic homeostasis through a negative feedback pathway in conditions of stress or aging. *Blood* 112, 4485–93. doi:10.1182/blood-2008-05-159848.

Molnarfi, N., Gruaz, L., Dayer, J.-M., and Burger, D. (2006). Opposite Regulation of IL-1 and Secreted IL-1 Receptor Antagonist Production by Phosphatidylinositide-3 Kinases in Human Monocytes Activated by Lipopolysaccharides or Contact with T Cells. *J. Immunol.* 178, 446–454. doi:10.4049/jimmunol.178.1.446.

Nagai, Y., Garrett, K. P., Ohta, S., Bahrun, U., Kouro, T., Takatsu, K., et al. (2006). Toll-like receptors on hematopoietic progenitor cells stimulate innate immune system replenishment. *Immunity* 24, 801–812.

Noh, K. T., Son, K. H., Jung, I. D., Kang, H. K., Hwang, S. A., Lee, W. S., et al. (2012). Protein kinase C δ (PKCδ)-extracellular signal-regulated kinase 1/2 (ERK1/2) signaling cascade regulates glycogen synthase kinase-3 (GSK-3) inhibition-mediated interleukin-10 (IL-10) expression in lipopolysaccharide (LPS)-induced endotoxemia. *J. Biol. Chem.* 287, 14226–14233. doi:10.1074/jbc.M111.308841.

Park, S., Guo, J., Kim, D., and Cheng, J. Q. (2008). Identification of 24p3 as a Direct Target of Foxo3a Regulated by Interleukin-3 through the Phosphoinositide 3-Kinase/Akt Pathway. *J. Biol. Chem.* 284, 2187–2193. doi:10.1074/jbc.M806131200.

Peled, A., Kollet, O., Ponomaryov, T., Petit, I., Franitza, S., Grabovsky, V., et al. (2000). The chemokine SDF-1 activates the integrins LFA-1, VLA-4, and VLA-5 on immature human CD34(+) cells: role in transendothelial/stromal migration and engraftment of NOD/SCID mice. *Blood* 95, 3289–96. Available at: http://www.ncbi.nlm.nih.gov/pubmed/10828007.

Perry, J. M., He, X. C., Sugimura, R., Grindley, J. C., Haug, J. S., Ding, S., et al. (2011). Cooperation between both Wnt/β-catenin and PTEN/PI3K/Akt signaling promotes primitive hematopoietic

stem cell self-renewal and expansion. *Genes Dev.* 25. doi:10.1101/gad.17421911.

Ponte, A. L., Ribeiro-Fleury, T., Chabot, V., Gouilleux, F., Langonné, A., Hérault, O., et al. (2012). Granulocyte-Colony-Stimulating Factor Stimulation of Bone Marrow Mesenchymal Stromal Cells Promotes CD34+ Cell Migration Via a Matrix Metalloproteinase-2-Dependent Mechanism. *Stem Cells Dev.* 21, 120724080005007. doi:10.1089/scd.2012.0048.

Reddy, S. a. G., Huang, J. H., and Liao, W. S.-L. (1997). Phosphatidylinositol 3-Kinase in Interleukin 1 Signaling: PHYSICAL INTERACTION WITH THE INTERLEUKIN 1 RECEPTOR AND REQUIREMENT IN NFkB AND AP-1 ACTIVATION. *J. Biol. Chem.* 272, 29167–29173. doi:10.1074/jbc.272.46.29167.

Sanz-Rodríguez, F., Hidalgo, A., and Teixidó, J. (2001). Chemokine stromal cell-derived factor-1alpha modulates VLA-4 integrin-mediated multiple myeloma cell adhesion to CS-1/fibronectin and VCAM-1. *Blood* 97, 346–351. doi:10.1182/blood.V97.2.346.

Satija, N. K., Sharma, D., Afrin, F., Tripathi, R. P., and Gangenahalli, G. (2013). High Throughput Transcriptome Profiling of Lithium Stimulated Human Mesenchymal Stem Cells Reveals Priming towards Osteoblastic Lineage. *PLoS One* 8. doi:10.1371/journal.pone.0055769.

Schajnovitz, A., Itkin, T., D'Uva, G., Kalinkovich, A., Golan, K., Ludin, A., et al. (2011). CXCL12 secretion by bone marrow stromal cells is dependent on cell contact and mediated by connexin-43 and connexin-45 gap junctions. *Nat. Immunol.* 12, 391–8. doi:10.1038/ni.2017.

Schofield, K., Humphries, M., de Wynter, E., Testa, N., and Gallagher, J. (1998). The Effect of α4β1-Integrin Binding Sequences of Fibronectin on Growth of Cells From Human Hematopoietic Progenitors. *Blood* 91, 3230–8.

Scott, L. M., Priestley, G. V, and Papayannopoulou, T. (2003). Deletion of alpha4 integrins from adult hematopoietic cells reveals roles in homeostasis, regeneration, and homing. *Mol. Cell. Biol.* 23, 9349–60. Available at: http://www.ncbi.nlm.nih.gov/pubmed/14645544 [Accessed June 29, 2016].

Semerad, C. L., Christopher, M. J., Liu, F., Short, B., Simmons, P. J., Winkler, I., et al. (2005). G-CSF potently inhibits osteoblast activity and CXCL12 mRNA expression in the bone marrow. *Blood* 106, 3020–7. doi:10.1182/blood-2004-01-0272.

Shankar, S., Marsh, L., and Srivastava, R. K. (2013). EGCG inhibits growth of human pancreatic tumors orthotopically implanted in Balb C Nude mice through modulation of FKHRL1/FOXO3a and neuropilin. *Mol. Cell. Biochem.* 372, 83–94. doi:10.1007/s11010-012-1448-y.EGCG.

Sharif-Askari, E., Vassen, L., Kosan, C., Khandanpour, C., Gaudreau, M., Heyd, F., et al. (2010). Zinc Finger Protein Gfi1 Controls the Endotoxin-Mediated Toll-Like Receptor Inflammatory Response by Antagonizing NF-kB p65 Zinc Finger Protein Gfi1 Controls the Endotoxin-Mediated Toll-Like Receptor Inflammatory Response by Antagonizing NF-kB. *Mol. Cell. Biol.*, 3929–3942. doi:10.1128/MCB.00087-10.

Sierro, F., Biben, C., Martínez-Muñoz, L., Mellado, M., Ransohoff, R. M., Li, M., et al. (2007). Disrupted cardiac development but normal hematopoiesis in mice deficient in the second CXCL12/SDF-1 receptor, CXCR7. *Proc. Natl. Acad. Sci. U. S. A.* 104, 14759–64. doi:10.1073/pnas.0702229104.

Silva, A., Yunes, J. A., Cardoso, B. a., Martins, L. R., Jotta, P. Y., Abecasis, M., et al. (2008). PTEN posttranslational inactivation and hyperactivation of the PI3K/Akt pathway sustain primary T cell leukemia viability. *J. Clin. Invest.* 118, 3762–3774. doi:10.1172/JCI34616.

Sizemore, N., Leung, S., and Stark, G. R. (1999). Activation of Phosphatidylinositol 3-Kinase in Response to Interleukin-1 Leads to Phosphorylation and Activation of the NF- kB p65/RelA Subunit. *Mol. Cell. Biol.* 19, 4798–4805.

Spiegel, A., Kollet, O., Peled, A., Abel, L., Nagler, A., Bielorai, B., et al. (2004). Unique SDF-1-induced activation of human precursor-B ALL cells as a result of altered CXCR4 expression and signaling. *Blood* 103, 2900–7. doi:10.1182/blood-2003-06-1891.

Sugiyama, T., Kohara, H., Noda, M., and Nagasawa, T. (2006). Maintenance of the hematopoietic stem cell pool by CXCL12-CXCR4 chemokine signaling in bone marrow stromal cell niches. *Immunity* 25, 977–88. doi:10.1016/j.immuni.2006.10.016.

Tabe, Y., Jin, L., Tsutsumi-Ishii, Y., Xu, Y., McQueen, T., Priebe, W., et al. (2007). Activation of integrin-linked kinase is a critical prosurvival pathway induced in leukemic cells by bone marrow-derived stromal cells. *Cancer Res.* 67, 684–694. doi:10.1158/0008-5472.CAN-06-3166.

Tak, P. P., and Firestein, G. S. (2001). NF- κB: a key role in inflammatory diseases. *J. Clin. Invest.* 107, 7–11.

Tamura, M., Sato, M. M., and Nashimoto, M. (2011). Regulation of CXCL12 expression by canonical Wnt signaling in bone marrow stromal cells. *Int. J. Biochem. Cell Biol.* 43, 760–7. doi:10.1016/j.biocel.2011.01.021.

Tarnowski, M., Liu, R., Wysoczynski, M., Ratajczak, J., Kucia, M., and Ratajczak, M. Z. (2010). CXCR7: A new SDF-1-binding receptor in contrast to normal CD34+ progenitors is functional and is expressed at higher level in human malignant hematopoietic cells. *Eur. J. Haematol.* 85, 472–483. doi:10.1111/j.1600-0609.2010.01531.x.

Tapia-Abellán, a., Ruiz-Alcaraz, a. J., Antón, G., Miras-López, M., Francés, R., Such, J., et al. (2014). Regulatory role of PI3K-protein kinase B on the release of interleukin-1β in peritoneal macrophages from the ascites of cirrhotic patients. *Clin. Exp. Immunol.* 178, 525–536. doi:10.1111/cei.12428.

Tjin, E. P. M., Groen, R. W. J., Vogelzang, I., Derksen, P. W. B., Klok, M. D., Meijer, H. P., et al. (2006). Functional analysis of HGF/MET signaling and aberrant HGF-activator expression in diffuse large B-cell lymphoma. *Blood* 107, 760–8. doi:10.1182/blood-2005-05-1929.

Torossian, F., Anginot, A., Chabanon, A., Clay, D., Guerton, B., Desterke, C., et al. (2014). CXCR7 participates in CXCL12-induced CD34+ cell cycling through b-arrestin–dependent Akt activation. 123, 191–202. doi:10.1182/blood-2013-05-500496.

Uto-Konomi, A., McKibben, B., Wirtz, J., Sato, Y., Takano, A., Nanki, T., et al. (2013). CXCR7 agonists inhibit the function of CXCL12 by down-regulation of CXCR4. *Biochem. Biophys. Res. Commun.* 431, 772–776. doi:10.1016/j.bbrc.2013.01.032.

Vagima, Y., Avigdor, A., Goichberg, P., Shivtiel, S., Tesio, M., Kalinkovich, A., et al. (2009). MT1-MMP and RECK are involved in human CD34+ progenitor cell retention, egress, and mobilization. *J. Clin. Invest.* 119, 492–503. doi:10.1172/JCI36541.

Tzeng, Y.-S., Li, H., Kang, Y.-L., Chen, W.-C., Cheng, W.-C., and Lai, D.-M. (2011). Loss of Cxcl12/Sdf-1 in adult mice decreases the quiescent state of hematopoietic stem/progenitor cells and alters the pattern of hematopoietic regeneration after myelosuppression. *Blood* 117, 429–39. doi:10.1182/blood-2010-01-266833.

Wang, L., You, L., Ni, W., Ma, Q., Tong, Y., Mao, L., et al. (2013). β-Catenin and AKT are promising targets for combination therapy in acute myeloid leukemia. *Leuk. Res.* 37, 1329–40. doi:10.1016/j.leukres.2013.06.023.

Wang, M. W., Consoli, U., Lane, C. M., Durett, A., Lauppe, M. J., Champlin, R., et al. (1998). Rescue from apoptosis in early (CD34-selected) versus late (non-CD34-selected) human hematopoietic cells by very late antigen 4- and vascular cell adhesion molecule (VCAM) 1-dependent adhesion to bone marrow stromal cells. *Cell Growth Differ.* 9, 105–12. Available at: http://www.ncbi.nlm.nih.gov/pubmed/9486846 [Accessed June 24, 2016].

Wang, T., Zou, J., Cunningham, C., and Secombes, C. J. (2002). Cloning and functional characterisation of the interleukin-1 beta 1 promoter of rainbow trout (Oncorhynchus mykiss). *Biochim. Biophys. Acta* 1575, 108–16. Available at: http://www.ncbi.nlm.nih.gov/pubmed/12020825.

Wang, Z. J., Zhang, F. M., Wang, L. S., Yao, Y. W., Zhao, Q., and Gao, X. (2009). Lipopolysaccharides can protect mesenchymal stem cells (MSCs) from oxidative stress-induced apoptosis and enhance proliferation of MSCs via Toll-like receptor(TLR)-4 and PI3K/Akt. *Cell Biol. Int.* 33, 665–674. doi:10.1016/j.cellbi.2009.03.006.

Williams, D. L., Li, C., Ha, T., Ozment-Skelton, T., Kalbfleisch, J. H., Preiszner, J., et al. (2004). Modulation of the phosphoinositide 3-kinase pathway alters innate resistance to polymicrobial sepsis. *J. Immunol.* 172, 449–456. doi:10.4049/jimmunol.172.1.449.

Xu, J., Qian, J., Xie, X., Lin, L., Zou, Y., Fu, M., et al. (2012). High density lipoprotein protects mesenchymal stem cells from oxidative stress-induced apoptosis via activation of the PI3K/Akt pathway and suppression of reactive oxygen species. *Int. J. Mol. Sci.* 13, 17104–17120. doi:10.3390/ijms131217104.

Yagisawa, M., Yuo, A., Kitagawa, S., Yazaki, Y., Togawa, A., and Takaku, F. (1995). Stimulation and priming of human neutrophils by IL-1 alpha and IL-1 beta: complete inhibition by IL-1 receptor antagonist and no interaction with other cytokines. *Exp. Hematol.* 23, 603–8. Available at: http://www.ncbi.nlm.nih.gov/pubmed/7601250 [Accessed August 21, 2015].

Yalcin, S., Zhang, X., Luciano, J. P., Mungamuri, S. K., Marinkovic, D., Vercherat, C., et al. (2008). Foxo3 is essential for the regulation of ataxia telangiectasia mutated and oxidative stress-mediated homeostasis of hematopoietic stem cells. *J. Biol. Chem.* 283, 25692–25705. doi:10.1074/jbc.M800517200.

Yang, J. C.-S., Wu, S.-C., Rau, C.-S., Lu, T.-H., Wu, Y.-C., Chen, Y.-C., et al. (2014). Inhibition of the phosphoinositide 3-kinase pathway decreases innate resistance to lipopolysaccharide toxicity in TLR4 deficient mice. *J. Biomed. Sci.* 21, 20. doi:10.1186/1423-0127-21-20.

Yang, J., Zong, C. S., Xia, W., Yamaguchi, H., Ding, Q. Q., Xie, X., et al. (2008). ERK promotes tumorigenesis by inhibiting FOXO3a via MDM2-mediated degradation. *Nat. Cell Biol.* 10, 138–148. doi:10.1038/ncb1676.

Yang, K., Wang, X. Q., He, Y. S., Lu, L., Chen, Q. J., Liu, J., et al. (2010). Advanced glycation end products induce chemokine/cytokine production via activation of p38 pathway and inhibit proliferation and migration of bone marrow mesenchymal stem cells. *Cardiovasc. Diabetol.* 9, 66. doi:10.1186/1475-2840-9-66.

Yücel, R., Kosan, C., Heyd, F., and Möröy, T. (2004). Gfi1: Green fluorescent protein knock-in mutant reveals differential expression and autoregulation of the growth factor independence 1 (Gfi1) gene during lymphocyte development. *J. Biol. Chem.* 279, 40906–17. doi:10.1074/jbc.M400808200.

Zeng, H., Yücel, R., Kosan, C., Klein-Hitpass, L., and Möröy, T. (2004). Transcription factor Gfi1 regulates self-renewal and engraftment of hematopoietic stem cells. *EMBO J.* 23, 4116–4125. doi:10.1038/sj.emboj.7600419.

Zhang, Y., Zhai, W., Zhao, M., Li, D., Chai, X., Cao, X., et al. (2015). Effects of Iron Overload on the Bone Marrow Microenvironment in Mice. *PLoS One* 10, e0120219. doi:10.1371/journal.pone.0120219.

Zhou, S. (2011). TGF-β regulates β-catenin signaling and osteoblast differentiation in human mesenchymal stem cells. *J. Cell. Biochem.* 112, 1651–1660. doi:10.1002/jcb.23079.TGF-.

Zhu, Z., Yin, J., Guan, J., Hu, B., Niu, X., Jin, D., et al. (2014). Lithium stimulates human bone marrow derived mesenchymal stem cell proliferation through GSK-3β-dependent β-catenin/Wnt pathway activation. *FEBS J.* 281, 5371–5389. doi:10.1111/febs.13081.

Zhuang, D., Qiu, Y., Kogan, S. C., and Dong, F. (2006). Increased CCAAT enhancer-binding protein ? (C/EBP?) expression and premature apoptosis in myeloid cells expressing Gfi-1 N382S mutant associated with severe congenital neutropenia. *J. Biol. Chem.* 281, 10745–10751. doi:10.1074/jbc.M510924200.
