# Supplementary material for: Modeling the Pro-inflammatory Tumor Microenvironment in Acute Lymphoblastic Leukemia Predicts a Breakdown of Hematopoietic-Mesenchymal Communication Networks
Source: Front Physiol. 2016 Aug 19;7:349. doi: 10.3389/fphys.2016.00349 (PMC4990565; doi:10.3389/fphys.2016.00349)
Supplement: Supplementary file 6 [file Image1.PDF]

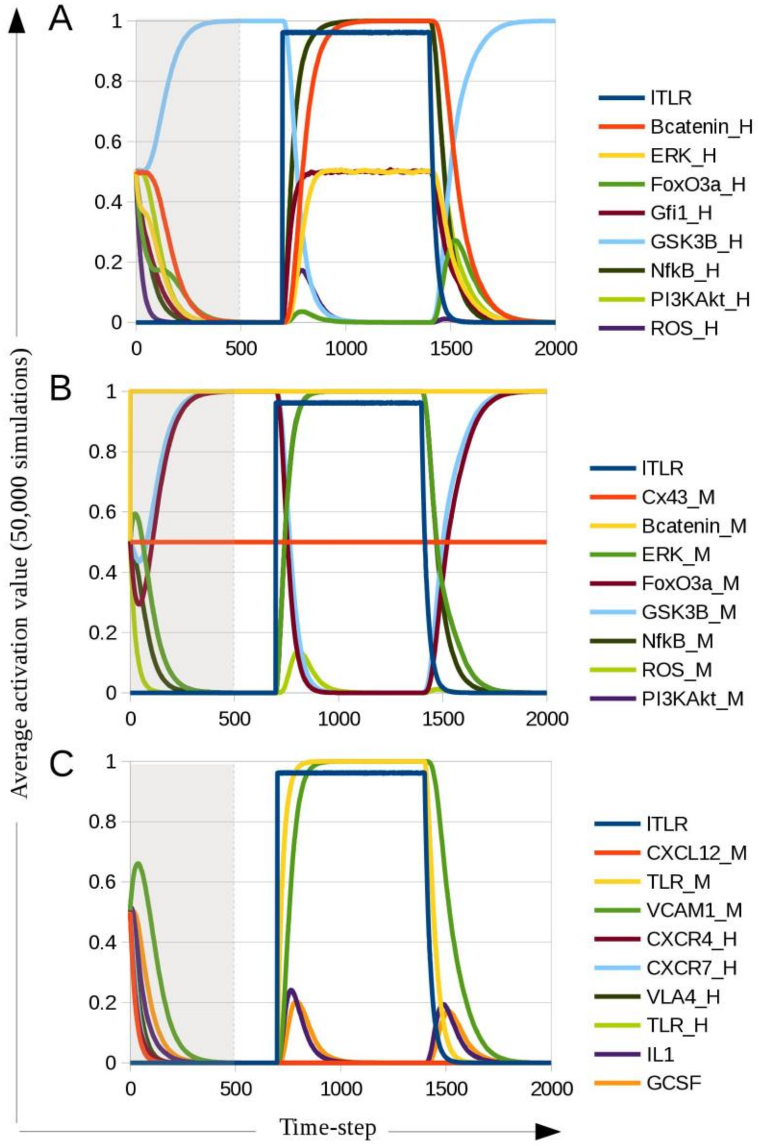

Figure S1. Dynamic multicellular approach for validation of lTLR-dependent VCAM-1 restoration induced by constitutive activation of β-catenin in MSC. Average activation value for intracellular HSPC nodes (A), intracellular MSC nodes (B) and communication axes among HSPC, MSC and microenvironment (C). lTLR was induced in time-step 700 and further sustained during 699 time-steps. Grey area covers the stabilization time steps until attractors are reached.
