# Supplementary material for: Modeling the Pro-inflammatory Tumor Microenvironment in Acute Lymphoblastic Leukemia Predicts a Breakdown of Hematopoietic-Mesenchymal Communication Networks
Source: Front Physiol. 2016 Aug 19;7:349. doi: 10.3389/fphys.2016.00349 (PMC4990565; doi:10.3389/fphys.2016.00349)
Supplement: Supplementary file 7 [file Image2.PDF]

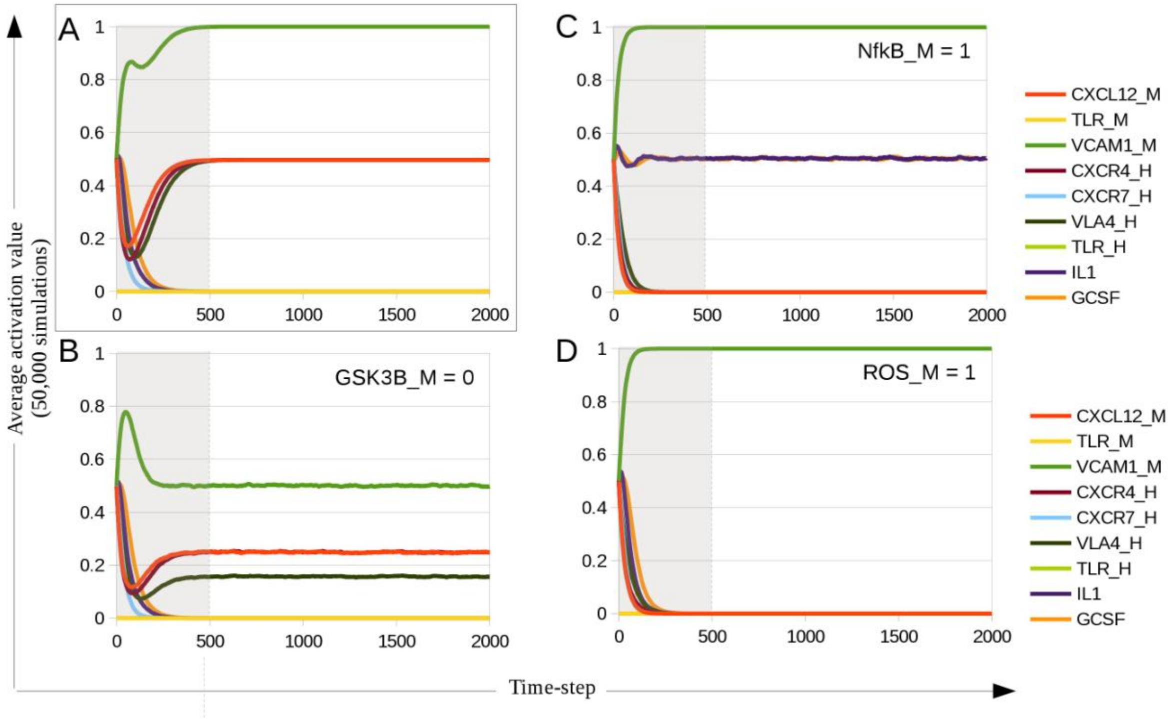


**Figure S2**. Dynamic multicellular simulation for wildtype network (A), GSK3β loss of function (B), NF-κB gain of function (C) and ROS overexpression in MSC. Average activation values for nodes involved in the intercellular communication axes are shown. Nodes representing molecules in HSPC are denoted with '_H' at the end of the node name, while nodes representing molecules in MSC are denoted with '_M'. Grey area covers the stabilization time steps until attractors are reached.
