# Supplementary material for: Modeling the Pro-inflammatory Tumor Microenvironment in Acute Lymphoblastic Leukemia Predicts a Breakdown of Hematopoietic-Mesenchymal Communication Networks
Source: Front Physiol. 2016 Aug 19;7:349. doi: 10.3389/fphys.2016.00349 (PMC4990565; doi:10.3389/fphys.2016.00349)
Supplement: Supplementary file 8 [file Image3.PDF]

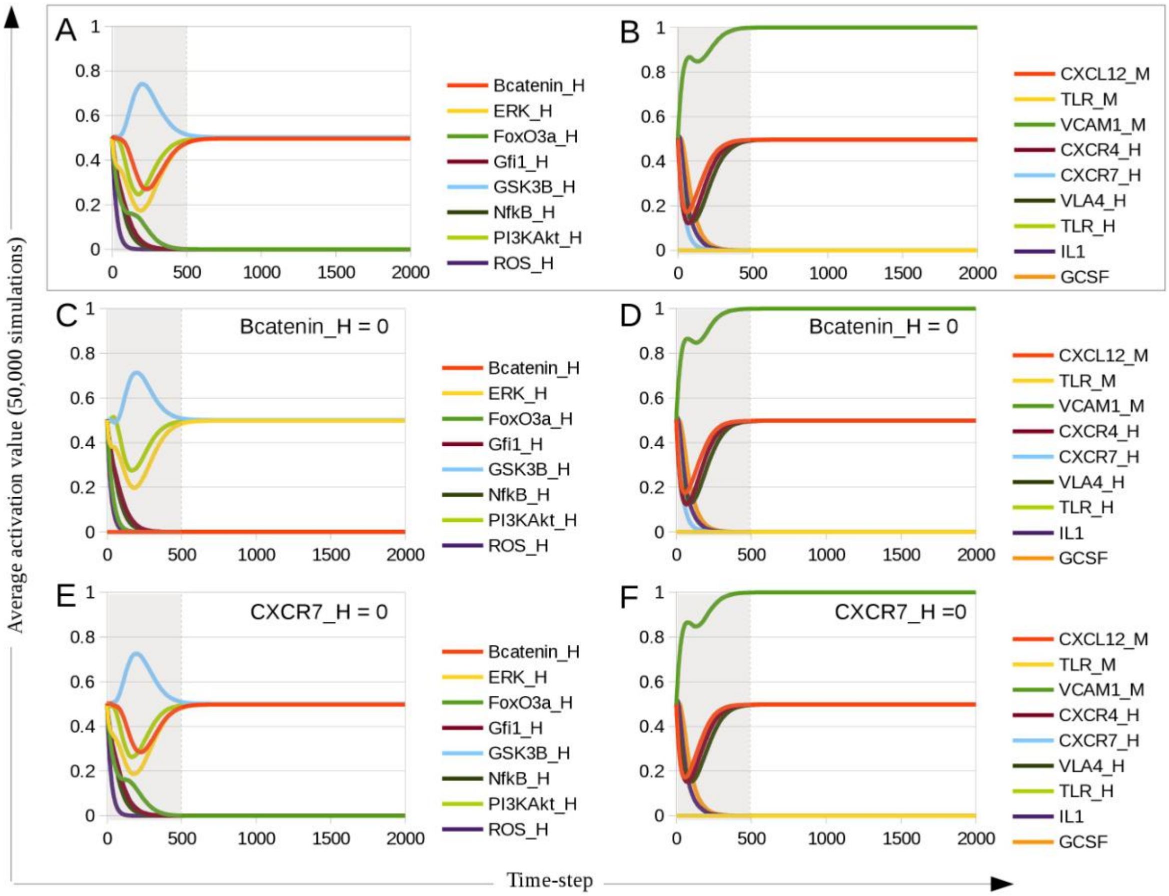

Figure S3. Dynamic multicellular simulation for wildtype network (A, B), β-catenin (C, D) and CXCR7 (E, F) null mutants in HSPC. Average activation values for the nodes involved in intracellular signaling of HSPC (A,C,E) and intercellular communication axes are shown (B, D, F). Nodes representing molecules in HSPC are denoted with '_H' at the end of the node name, while nodes representing molecules in MSC are denoted with '_M'. Grey area covers the stabilization time steps until attractors are reached.
