# Supplementary material for: Modeling the Pro-inflammatory Tumor Microenvironment in Acute Lymphoblastic Leukemia Predicts a Breakdown of Hematopoietic-Mesenchymal Communication Networks
Source: Front Physiol. 2016 Aug 19;7:349. doi: 10.3389/fphys.2016.00349 (PMC4990565; doi:10.3389/fphys.2016.00349)
Supplement: Supplementary file 9 [file Image4.PDF]

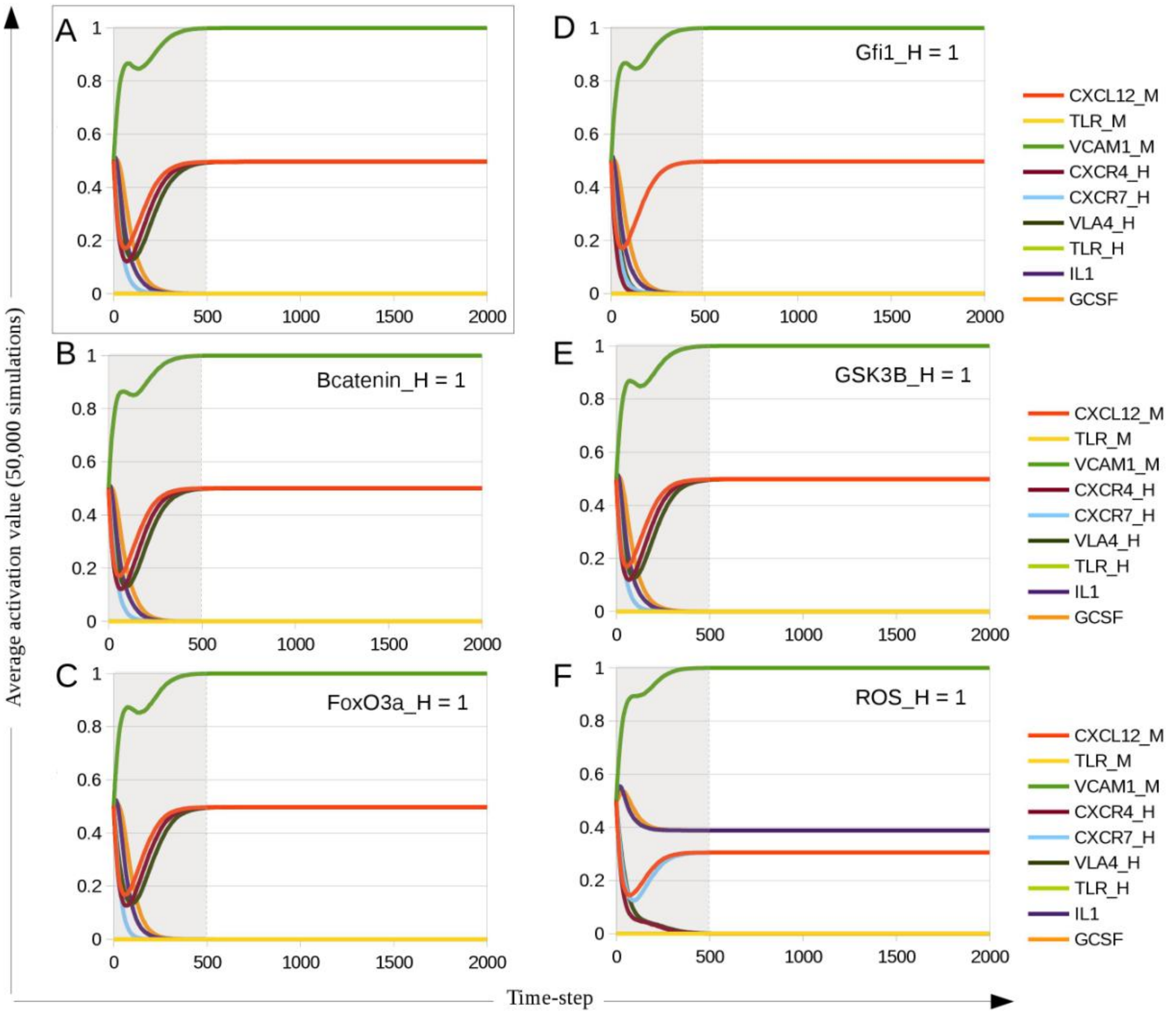
A
B
Bcatenin_H = 1
C
FoxO3a_H = 1
D
Gfi1_H = 1
E
GSK3B_H = 1
F
ROS_H = 1
CXCL12_M
TLR_M
VCAM1_M
CXCR4_H
CXCR7_H
VLA4_H
TLR_H
IL1
GCSF
Average activation value (50,000 simulations)
Time-step
0
0.2
0.4
0.6
0.8
1
500
1000
1500
2000

Figure S4. Dynamic multicellular simulation for wildtype network (A) and β-catenin (B), FoxO3a (C), Gfi1 (D), GSK3β (E) and ROS (F) overexpression in HSPC. Average activation values for the nodes involved in the intercellular communications axes are shown. Nodes representing molecules in HSPC are denoted with '_H' at the end of the node name, while nodes representing molecules in MSC are denoted with '_M'. Grey area covers the stabilization time steps until attractors are reached.
