# Supplementary material for: Modeling the Pro-inflammatory Tumor Microenvironment in Acute Lymphoblastic Leukemia Predicts a Breakdown of Hematopoietic-Mesenchymal Communication Networks
Source: Front Physiol. 2016 Aug 19;7:349. doi: 10.3389/fphys.2016.00349 (PMC4990565; doi:10.3389/fphys.2016.00349)
Supplement: Supplementary file 10 [file Image5.PDF]

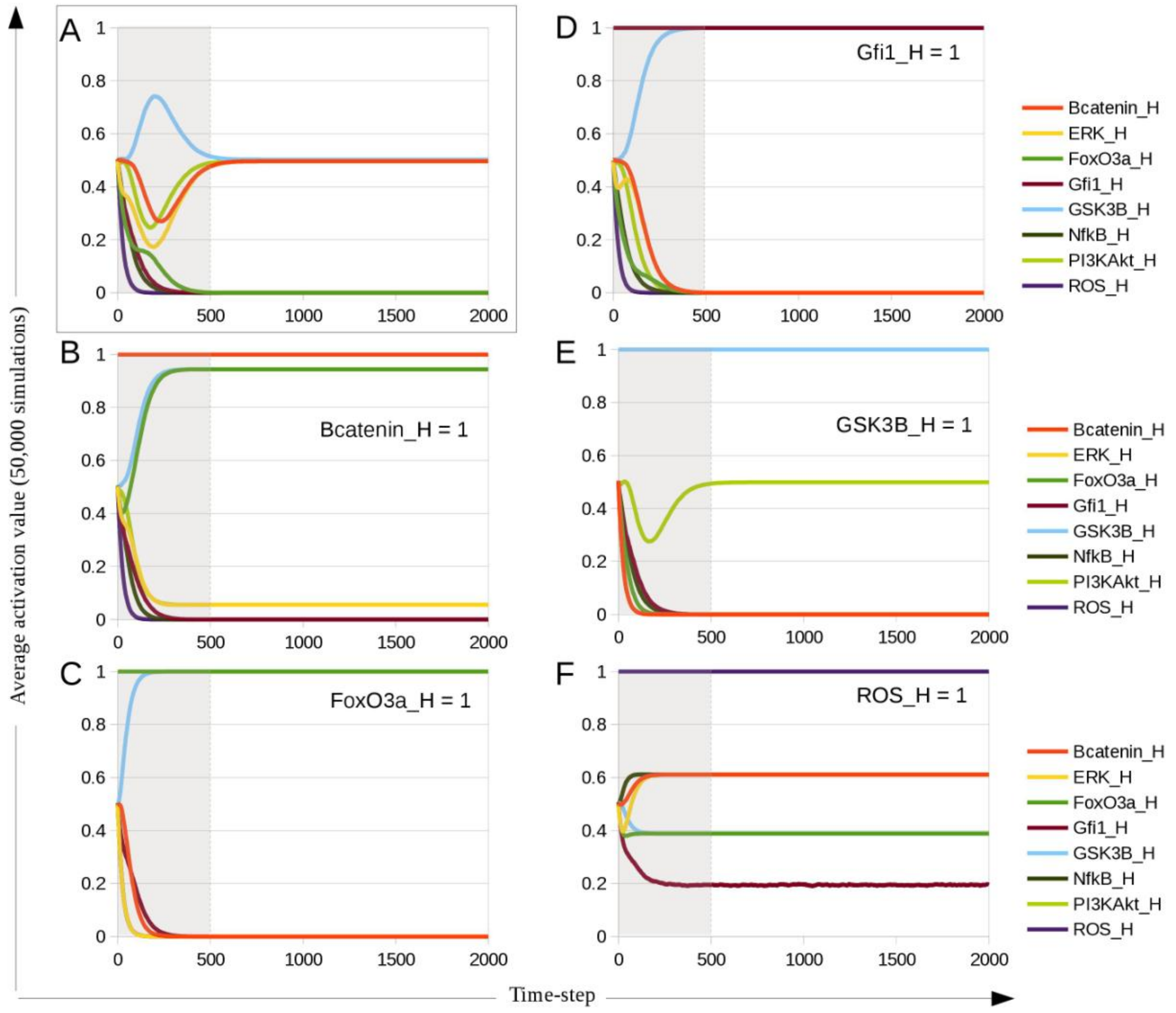

Average activation value (50,000 simulations)
Time-step
A
B
Bcatenin_H = 1
C
FoxO3a_H = 1
D
Gfi1_H = 1
E
GSK3B_H = 1
F
ROS_H = 1
0
0.2
0.4
0.6
0.8
1
0
500
1000
1500
2000
Bcatenin_H
ERK_H
FoxO3a_H
Gfi1_H
GSK3B_H
NfkB_H
PI3KAkt_H
ROS_H

Figure S5. Dynamic multicellular simulation for wildtype network (A) and β-catenin (B), FoxO3a (C), Gfi1 (D), GSK3β (E) and ROS (F) overexpression in HSPC. The average activation values for nodes involved in the intracellular signaling of HSPC are shown. Nodes representing molecules in HSPC are denoted with '_H' at the end of the node name, while nodes representing molecules in MSC are denoted with '_M'. Grey area covers the stabilization time steps until attractors are reached.
