# Supplementary material for: Modeling the Pro-inflammatory Tumor Microenvironment in Acute Lymphoblastic Leukemia Predicts a Breakdown of Hematopoietic-Mesenchymal Communication Networks
Source: Front Physiol. 2016 Aug 19;7:349. doi: 10.3389/fphys.2016.00349 (PMC4990565; doi:10.3389/fphys.2016.00349)
Supplement: Supplementary file 11 [file Image6.PDF]

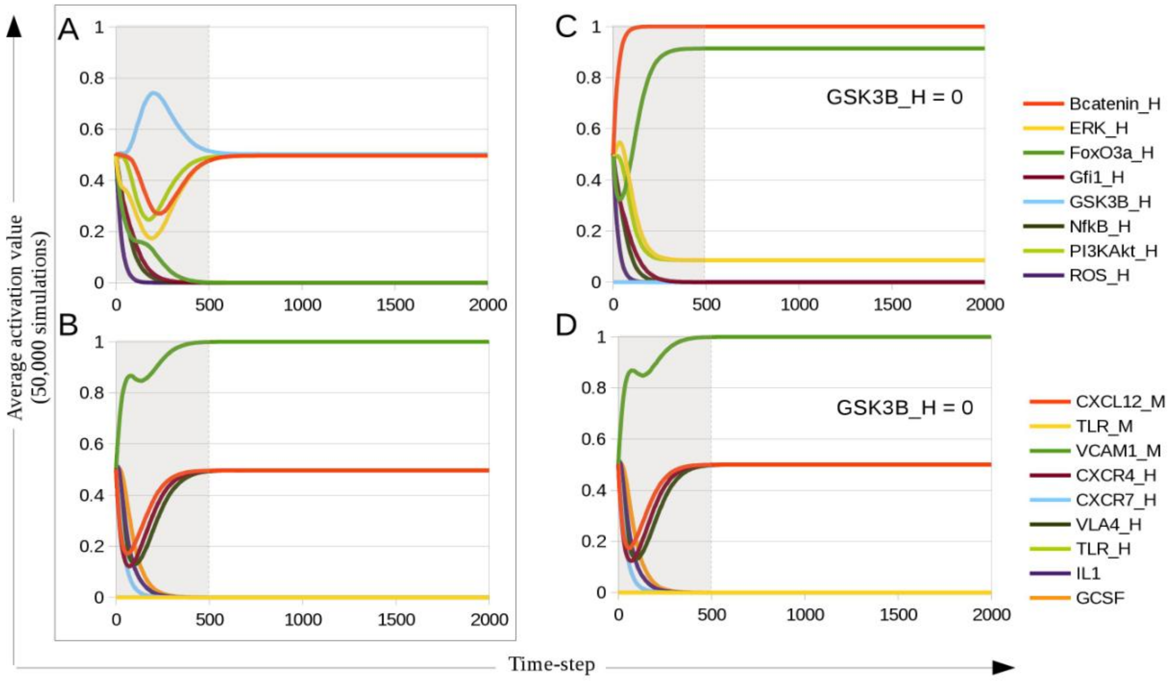
A
B
C
D
GSK3B_H = 0
GSK3B_H = 0
Average activation value
(50,000 simulations)
Time-step
Bcatenin_H
ERK_H
FoxO3a_H
Gfi1_H
GSK3B_H
NfkB_H
PI3KAkt_H
ROS_H
CXCL12_M
TLR_M
VCAM1_M
CXCR4_H
CXCR7_H
VLA4_H
TLR_H
IL1
GCSF

Figure S6. Dynamic multicellular simulation for wildtype network (A, B) and GSK3β (C, D) loss of function in HSPC. Average activation values for the nodes involved in the intracellular signaling of HSPC (A, C) and intercellular communication axes are shown (B, D). Nodes representing molecules in HSPC are denoted with '_H' at the end of the node name, while nodes representing molecules in MSC are denoted with '_M'. Grey area covers the stabilization time steps until attractors are reached.
